# Stiff-Stilbene-Linked Bis-Cholesterol: Synthesis and Investigation of Its Supramolecular Gelation and Photophysical Behaviors

**DOI:** 10.1021/acsomega.4c10136

**Published:** 2024-12-26

**Authors:** Dagninet Yeshiwas Alene, Wen-Sheng Chung

**Affiliations:** Department of Applied Chemistry, National Yang Ming Chiao Tung University, Hsinchu 30050, Taiwan, ROC

## Abstract

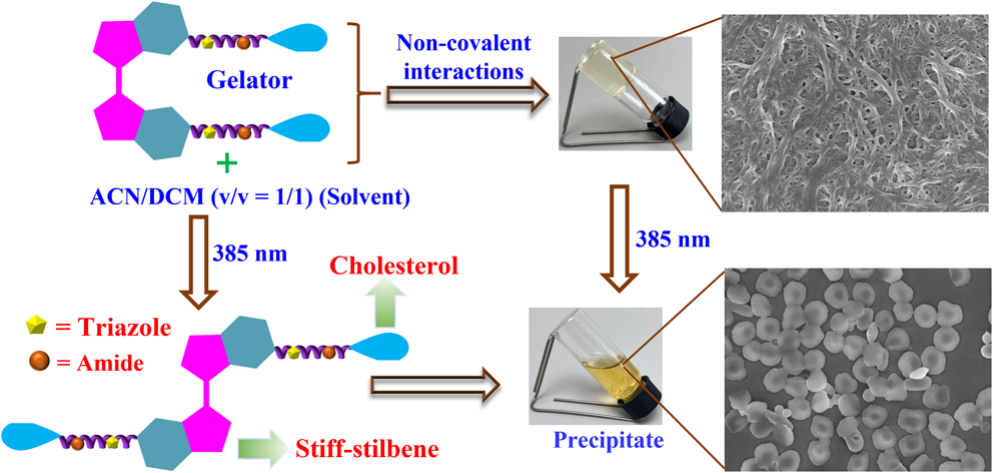

Here, we report the design, synthesis, and comprehensive characterization of the bis-cholesterol supramolecular gelator, which contains photochromic stiff-stilbene as a bridging unit. The *cis*-isomer of stiff-stilbene bridged bis-cholesterol (*Z*-**D**) was first synthesized with a systematic design, which can be further converted into its *trans*-isomer (*E*-**D**) with a high degree of efficiency (ca. 100%) upon exposure to 385 nm UV light. Unusual gelation behavior was observed for *Z*-**D**, which exhibited supergelator properties in mixed solvents of acetonitrile (ACN)/dichloromethane (DCM) (v/v = 1:1), DCM/MeOH (v/v = 1:2), ACN/CHCl_3_ (v/v = 2:1), and CHCl_3_/MeOH (v/v = 1:2), with minimum gelation concentrations (MGCs) as low as 0.2 w/v%. These gels formed rapidly at room temperature without the aid of any mechanical forces upon the addition of an antisolvent into the vial containing the gelator and its dissolving solvent. The formation of the self-assembled gel was primarily driven by hydrogen bonding, van der Waals forces, and dipole–dipole interactions, as confirmed by ^1^H NMR, Fourier transform infrared spectroscopy (FT-IR), and UV–vis spectroscopies. The gelator molecule *Z*-**D** entraps organic solvents and organizes itself into three-dimensional (3D) fibrillar networks in various single and mixed solvents, as confirmed by scanning electron microscopy (SEM) analysis. Upon irradiation with 385 nm light, the gel networks disintegrated into a precipitate suspension, resulting in the transformation of the fibrous structures into irregular spherical-like aggregates. This proves that the structural conformation changes in the gelator significantly influence the resulting self-assembled structures. Overall, the findings present in this study pave the way for the future development of novel light-responsive bis-cholesterol-based gelators, especially in their *Z*-isomeric form.

## Introduction

Stimuli-responsive supramolecular architectures have received considerable attention because they provide promising possibilities for the development of smart soft materials with dynamic characteristics and functions.^1−3^ Supramolecular gels, a class of soft materials formed through the noncovalent self-aggregation of low-molecular weight gelator molecules that immobilize a vast volume of solvent, have gained increasing attention over the past few decades due to their simple structure and dynamic functionality.^4−8^ The design and synthesis of novel gelator moieties through the integration of specific features are important tasks. External stimuli, which can be chemical (e.g., pH and redox) or physical (e.g., light and temperature), are employed to modulate the assembly and responsive behavior of gelators.^4,6,9,10^ Although numerous studies have been conducted on stimuli-responsive supramolecular gels, the design and synthesis of light-responsive supramolecular systems and the use of light as a remote control to modulate their morphology, functionality, self-aggregation, and disassembly remain challenging and vibrant areas of research. Compared to other physical and chemical stimuli, light is widely recognized as one of the most versatile stimuli, offering distinct advantages such as being nondestructive and not generating waste products in the system.^11−15^ Our research group has actively contributed to this field by developing various light-responsive supramolecular gelators.^16−19^ Recently, we reported a phototriggered low-molecular-mass organic gelator (LMOG) constructed with a light-sensitive stiff-stilbene moiety covalently linked to a *t*-butylcalix[4]arene macrocycle.^16^ Among photochromic molecules, stiff-stilbene offers several advantages, including large geometric changes upon isomerization, and a substantial activation barrier (ca. 43 kcal/mol) between its *Z* and *E* isomers, which makes it thermally stable and prevents thermal relaxation.^20^ As mentioned above, over the past few decades, extensive research has been conducted on the modulation of supramolecular systems in response to various stimuli,^6,7,21−29^ particularly focusing on the assembly and disassembly of aggregates (e.g., gel–sol transitions and morphological changes). The driving force for this self-organization process is often a combination of noncovalent interactions, including hydrogen bonding, π–π stacking, van der Waals forces, dipole–dipole interactions, and others.^30^ The field of stimuli-responsive low-molecular-weight supramolecular gels has witnessed significant growth and interest, driven by their diverse applications span various sectors, including medicine (e.g., drug delivery),^31^ environmental remediations (e.g., oil spill recovery),^32^ molecular motors,^33^ chemosensing,^34^ and other applications.^35−37^ However, among the various stimuli, investigation of light effects on gel–sol transitions and morphological changes within supramolecular systems is comparatively limited and remains challenging.

Cholesterol-functionalized LMOGs are a versatile class of self-assembling materials that have gained substantial research attention.^38^ It is known that functional cholesterol-based LMOGs were reported first by Weiss and co-workers more than 3 decades ago,^30,39^ and continue to be a subject of active research. This is because the cholesteryl groups tend to organize themselves in solution and form a self-assembled structure via weak van der Waals’ interaction.^40,41^ These forces are not strong but sufficient to cause the molecules to organize themselves in a regular pattern. A wide range of cholesterol-containing supramolecular systems that showed gelation behavior under various conditions have been reported^38,42−45^ and are exploited for various applications, including environmental remediation^46,47^ and selective separation of organic solvents with comparable polarity.^48^ However, based on our literature survey, no investigations of stiff-stilbene-bridged bis-cholesterol molecules have been reported as potential supramolecular gelators. Drawing inspiration from previous research, we designed and synthesized a novel bis-cholesterol derivative linked by a rigid stilbene unit that showed unusual supramolecular gelation behaviors. The integration of a photoactive stiff-stilbene unit into bis-cholesterol derivative endows photoswitching capabilities to the resulting supramolecular system. This study investigated the light-triggered self-assembly and disassembly behavior of a stiff-stilbene-linked bis-cholesterol supramolecular system, including its gelation behavior, configurational changes, and morphological transitions.

## Results and Discussion

### Molecular Design and Synthesis Approaches

**Scheme 1:**
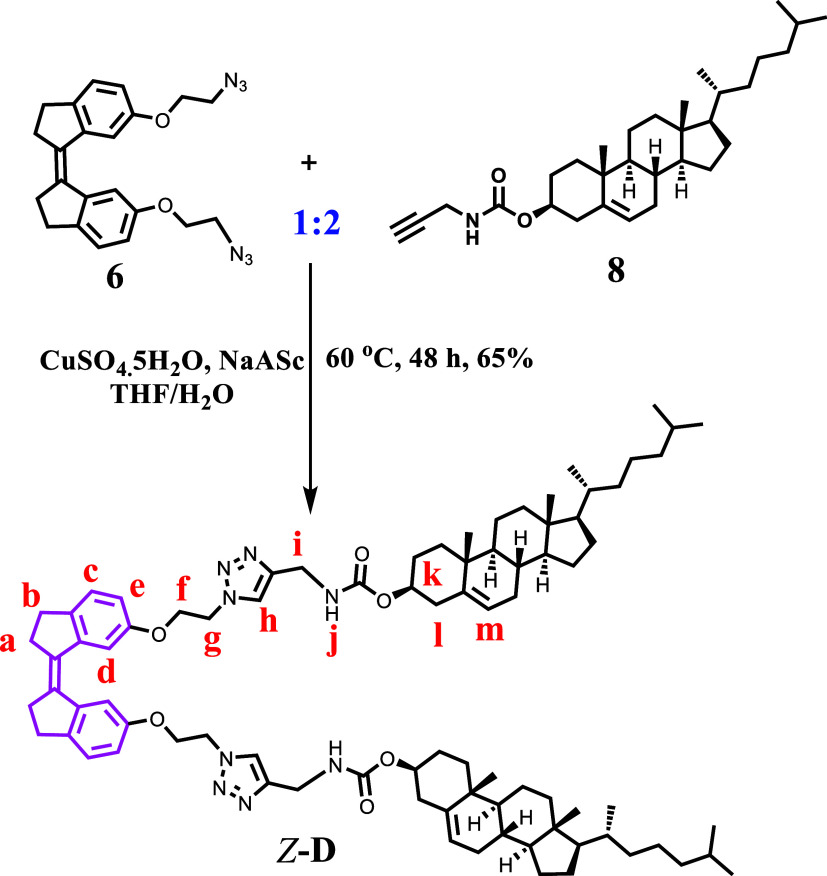
Synthesis of Compound *Z***-D**

This study aims to design and construct a light-responsive supramolecular system capable of trapping organic solvents and forming organogels. The target molecule *Z*-**D**, a bis-cholesterol derivative bridged by a photochromic stiff-stilbene unit, was designed and synthesized using a Click chemistry approach. This was achieved through Cu(I)-catalyzed azide–alkyne cycloaddition (CuAAC) reaction between a 1:2 ratio of diazido stiff-stilbene **6** and monopropargyl-terminated cholesterol **8**, yielding 65%, as shown in Scheme 1. Initially, the synthesis of two crucial building blocks, namely, a light-active *Z*-bisazido stiff-stilbene (**6**) and one side of a propargyl-terminated cholesterol derivative (**8**), was carried out. The photoactive center of a single isomer **6** was established via a systematic approach with a yield of 81% following our previously reported method and others,^16,49^ whereas compound **8** was synthesized as described in literature protocols.^50,51^ The synthetic pathways, procedures, and detailed characterization data for all compounds reported in this study are outlined in the Supporting Information (SI). The structures of newly synthesized compounds were elucidated using a combination of ^1^H NMR, ^13^C NMR, and high-resolution mass spectrometry (HRMS), and the corresponding spectra are presented in Figures S8–S14 (SI) for further structural verification. Furthermore, as shown in Figure S14a,b (SI), the structural elucidation (the assignment of proton environments) for the target molecules *Z*-**D** and *E*-**D** was confirmed through 2D H, homonuclear correlation spectroscopy (H-COSY) experiments. The results provide valuable insight into the proton–proton coupling interactions within the molecules. Apart from ^1^H NMR, ^13^C NMR, and high-resolution mass spectrometry (HRMS), Fourier-transform infrared (FT-IR) spectroscopy was utilized to monitor the progress of the click reaction between compounds **6** and **8**. The distinctive strong azide stretching vibration peak at 2104 cm^–1^, as shown in Figure S1 (SI), was eliminated following the click reaction. This disappearance indicates the consumption of the azide group in compound **6**, providing evidence for the formation of the desired *Z*-**D** product.

## Investigation of Photoswitching Properties

**Figure 1:**
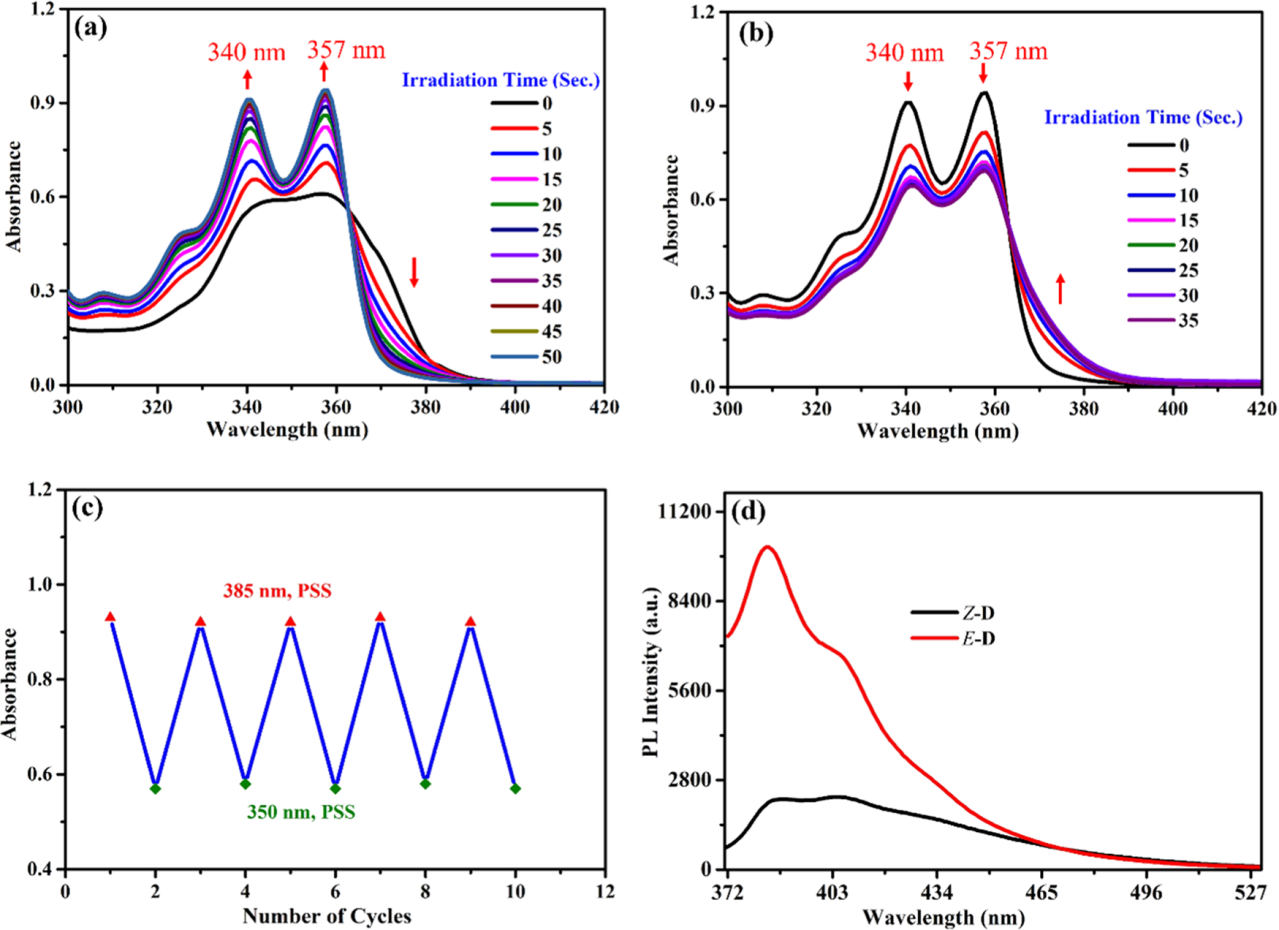
Photochemical isomerization and absorbance changes of compounds *Z***-D** and *E*-**D** (30 μM in degassed CH_2_Cl_2_): (a) Irradiation with 385 nm UV light for 50 s (*Z* → *E*), (b) irradiation of the solution at the photostationary state (PSS) with 350 nm UV light for 35 s (*E* → *Z*), and (c) the absorbance with repeated and alternative 385/350 nm irradiation cycles at the PSSs (50/35 s, respectively); (d) fluorescence spectra of *Z***-D** and *E*-**D**, λ_ex_ = 350 nm for *Z*- and 357 nm for *E*-structures.

**Figure 2:**
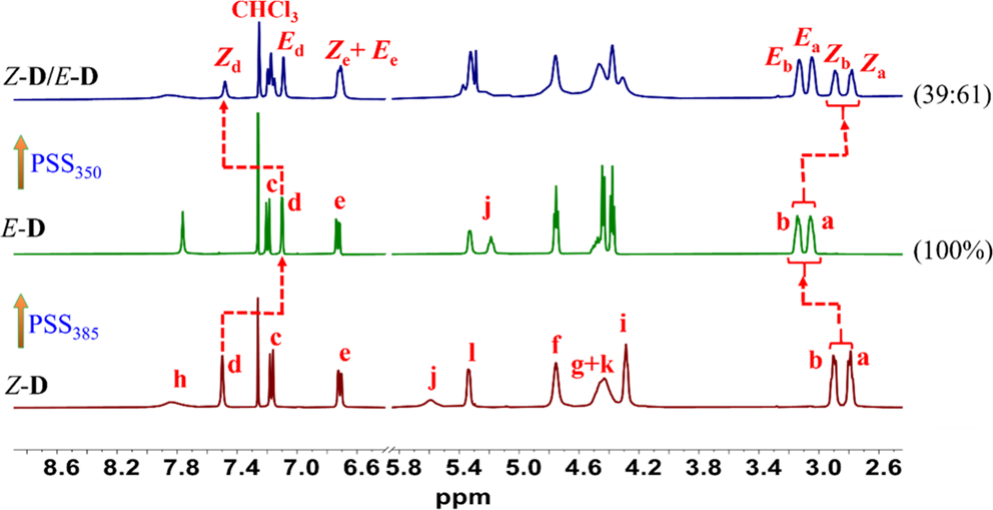
Partial ^1^H NMR spectral changes (CDCl_3_, 400 MHz, 298 K) upon consecutive irradiation of *Z*-**D** and *E*-**D** (1.2 mM in CH_2_Cl_2_) with 350 and 385 nm light at their PSS.

**Scheme 2:**
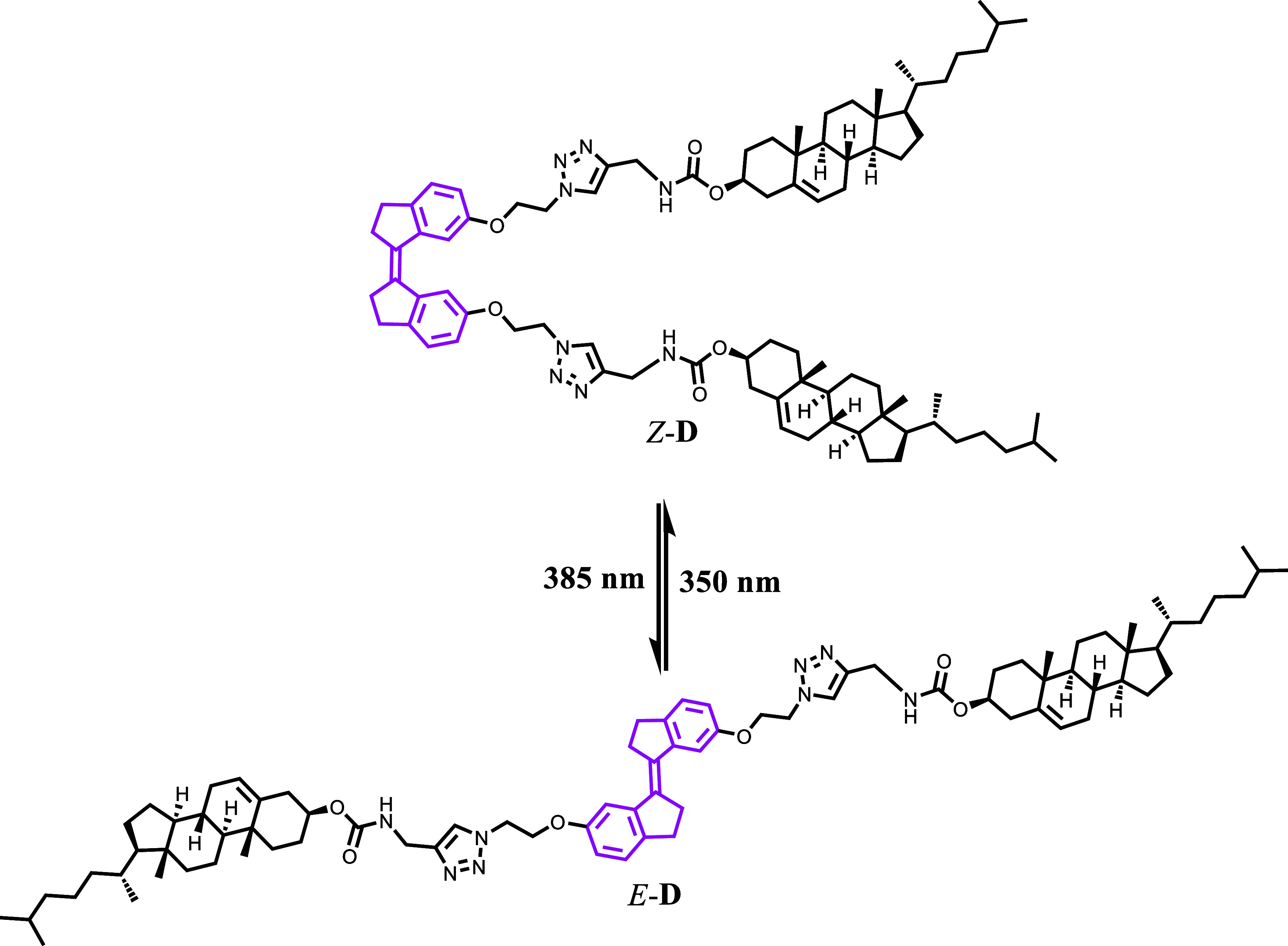
Photo-isomerization of *Z*-**D** to *E*-**D** and Its Reversible Process

The light source from an light-emitting diode (LED) lamp was used as a remote trigger for *Z*–*E* isomerization. The photoisomerization characteristics were investigated by using UV–vis and ^1^H NMR spectroscopies. As described above, a single isomer of *Z-*stiff-stilbene bridged bis-cholesterol (*Z*-**D**) was synthesized. Consequently, as shown in Scheme 2, Figures 1 and 2, it undergoes efficient photoisomerization to its nongelator *E*-counterpart (*E*-**D**) with quantitative conversion (ca. 100%) upon irradiation with 385 nm UV light in a degassed CH_2_Cl_2_ solution (30 μM). This method is reagent-free, requires a short conversion time, offers quantitative yield, and does not result in the formation of any side products or waste within the system.

Exposure of *Z*-**D** to 385 nm UV light (LED lamp) induced the emergence of new absorption bands at 340 and 357 nm, which progressively increased in intensity, as shown in Figure 1a. After 50 s of irradiation, no further changes in the intensities of these absorption bands were observed. This suggests that the photostationary state (PSS_385_) had been reached and prolonged irradiation beyond this duration did not result in any noticeable changes. The occurrence of this phenomenon indicates the switching of stiff-stilbene from the *Z*-isomer to its *E*-isomer counterpart.

To understand the dynamic behavior of switching between *E* and *Z* forms in response to light energy and the stability of the molecular system bis-cholesterol **D**, conducting photoisomerization reversibility studies is essential. When the resulting *E* solution at its PSS_385_ was exposed to 350 nm light for 35 s, it underwent a reverse *E*-to-*Z* isomerization, decreasing the maximum absorbance intensities at 340 and 357 nm, as illustrated in Figure 1b. The observation of an isosbestic point at a wavelength of 363 nm indicates that the isomerization transition is unimolecular.

Moreover, upon repeated alternating irradiation with 385 and 350 nm UV light, the molecular system **D** switched reversibly between its *Z* and *E* isomeric forms for at least five cycles, without exhibiting any significant fatigue phenomenon, as shown in Figure 1c. The photochemical switching of the bis-cholesterol **D** system resulted in significant differences in fluorescence emission intensity between the *Z* and *E* isomers, as presented in Figure 1d. In these spectra, the *Z*-**D** isomer exhibited a characteristic of weak and broadband fluorescence emission within the 371–500 nm range. Upon irradiation with 385 nm light, an enhanced emission at 384 nm was observed, indicating the conversion of *Z*-**D** to the *E*-**D** isomer, which adopts a more rigid and planar structure compared to its *Z* counterpart.

A ^1^H NMR experiment was performed (see Figure 2) to further investigate the reversible *Z*/*E* isomerization detected by UV–vis spectroscopy. It plays a crucial role in quantifying the conversion efficiency between light-induced *Z*–*E* isomerization of stiff-stilbene. Upon irradiation of the *Z*-**D** isomer at 385 nm, the ^1^H NMR spectrum exhibited a new set of signals with a complete shift, indicating a 100% conversion efficiency. The proton signal of H_d_, located at the core of stiff-stilbene, showed a significant upfield shift from 7.50 to 7.10 ppm (Δδ = −0.4 ppm). In contrast, all other aromatic stiff-stilbene proton signals underwent a downfield shift, indicating the occurrence of *Z*-to-*E* photoisomerization. Subsequent irradiation at 350 nm resulted in 39% recovery of the initial ^1^H NMR spectrum (*Z*-**D**), and 61% of the *E*-**D** isomer at the PSS was unconverted. The 39:61 conversion ratio of the *Z* and *E* isomers was determined from the ^1^H NMR integrals of peaks labeled as a–e, using the equation described in the Supporting Information.

## Gelation Studies

The presence of a hydrophobic cholesterol moiety, amide functional groups, triazole rings, and an aromatic stiff-stilbene at the center, which serves as a linker between the two cholesterol moieties, prompted us to investigate the gelation capabilities of *Z*-**D** in a range of various organic solvents, as summarized in Tables 1 and S1 (SI). The detailed gelation protocol is described in the Experimental Section of the Supporting Information. Bis-cholesterol *Z*-**D** isomer was freely soluble in solvents such as chloroform, dichloromethane, and tetrahydrofuran (THF). Gelation tests were performed both in mixed and single solvents, of which gels of mixed solvents were formed rapidly without the support of any external forces upon the addition of an antisolvent into the vial containing the gelator and its dissolving solvent. This suggests that the combined solvents provide an optimal environment for gelation, likely due to favorable solvent–gelator interactions. In contrast, *E*-**D** was soluble only in chloroform and THF but was insoluble in many of the solvents tested. The gelator uniquely forms a gel in its *Z*-isomer, unlike most molecules undergoing *Z*–*E* isomerization induced by light or other stimuli, which typically fail to gel in this conformation due to insufficient planarity for self-aggregation. However, in this work, *Z*-stiff-stilbene-linked bis-cholesterol has been proven to be an efficient supramolecular gelator. This efficiency arises from the strong tendency of the cholesteryl groups to organize themselves through cholesterol–cholesterol interactions, driven by weak van der Waals forces.^45^ These organized structures are further interconnected via hydrogen bonding and dipole–dipole interactions through the amide and triazole groups, respectively, resulting in the formation of three-dimensional (3D) fibrillar networked structures. Cholesterol groups with a folded linker structure can undergo self-aggregation in many gels driven by van der Waals forces, resulting in a helical stacking arrangement.^45,52,53^ The folded structure is expected to exhibit efficient intramolecular and intermolecular cholesterol–cholesterol, amide–amide of the carbamate, and triazole–triazole interactions. Moreover, in its *Z* conformation, the cholesterol units are in closer proximity to one another, enabling them to effectively trap and immobilize solvent molecules, thereby facilitating gel formation. The gelation potential of *Z*-**D** in mixed polar and nonpolar organic solvents was ascertained during the process of examining its ability to generate single crystals using various solvent combinations. Despite testing multiple solvents, *Z*-**D** did not form single crystals in any of the solvents due to its high amorphous characteristics. Instead, it demonstrated gelation capability in mixed solvents of acetonitrile (ACN)/dichloromethane (DCM) (v/v = 1:1), ACN/CHCl_3_ (v/v = 2:1), DCM/MeOH (v/v = 1:2), and CHCl_3_/MeOH (v/v = 1:2). The *Z*-isomer, *Z*-**D**, also showed partial solubility in nonpolar organic solvents such as toluene, benzene, and *p*-xylene at room temperature. However, when heated with a heat gun, it completely dissolved and, subsequently, transformed into a gel upon cooling to room temperature. The results suggested that thermal energy supplied is required to overcome the activation barrier for gelation in these solvents. The minimum gelation concentration (MGC) of *Z*-**D** ranges from 0.2 w/v% in mixed solvent systems such as ACN/DCM, DCM/MeOH, ACN/CHCl_3_, and CHCl_3_/MeOH to 1.8 w/v% in a single solvent, benzene. This indicates that *Z*-**D** can be classified as a supergelator of the aforementioned mixed solvents.^54^

**Table 1:** Gelation Properties of *Z***-D** and the Critical Gelation Concentrations (w/v%) in Various Organic Solvents^a^

	states	
solvents	*Z*-**D**	*E*-**D**
toluene	G (0.8)	P
DCM/MeOH (v/v = 1:2)	G (0.2)	P
CHCl_3_/MeOH (v/v = 1:2)	G (0.2)	P
ACN/DCM (v/v = 1:1)	G (0.2)	P
ACN/CHCl_3_ (v/v = 2:1)	G (0.2)	P

a P = precipitates G = gel; numbers in brackets represent their MGC values in w/v% = [(g/100 mL) %].

The minimum gelation concentration (MGC) values were determined by the successive addition of solvent to the original gel system. The corresponding photographic images of gels shown in Figure S2a (SI) are stable at room temperature for several weeks. The *Z*-**D** gels obtained in various solvents exhibited reversible sol-to-gel transitions in response to temperature changes, as demonstrated for the ACN/DCM (v/v = 1:1) gel in Figure S2b (SI), indicating the presence of a supramolecular gel network. This behavior is typically due to the disruption and reformation of noncovalent interactions such as hydrogen bonding, van der Waals forces, and dipole–dipole interactions within the gel structure.

## Investigation of Driving Forces for Gelation

**Figure 3:**
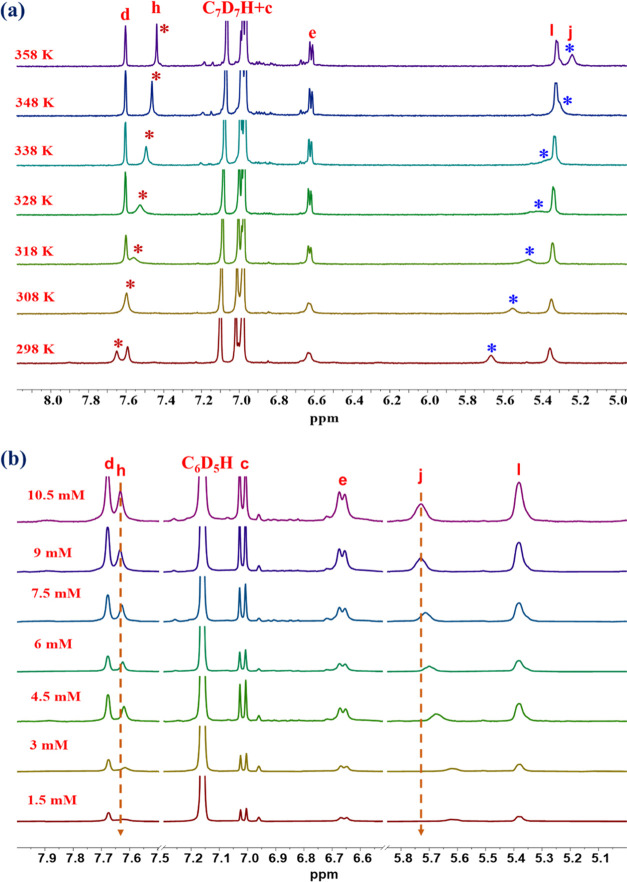
Partial ^1^H NMR spectra of *Z*-**D**: (a) temperature-dependent in toluene-*d*_8_ at a concentration of 1.5 w/v%, and (b) concentration-dependent spectra in benzene-*d*_6_.

It is widely recognized that the formation of supramolecular gels is driven by various noncovalent interactions, which typically result in a reversible gel-to-sol phase transition. One of the effective approaches to investigate the potential noncovalent interaction formations during the gelation process is ^1^H NMR spectroscopy. To elucidate the possible driving forces behind the gelation, temperature-dependent ^1^H NMR measurements of the *Z*-**D** gel system in toluene-*d*_8_ (1.5 w/v%) were performed, and the results are presented in Figure 3a. Upon increasing the temperature from 298 to 358 K, the signals of the amide proton −NH (H_j_) shifted from 5.7 to 5.2 ppm and the triazole proton (H_h_) also exhibited upfield shifts from 7.7 to 7.4 ppm, indicating the presence of hydrogen bonding and dipole–dipole interactions, respectively. The rest of the other proton signals showed relatively little changes in the temperature range. These changes suggest the disruption of hydrogen bonding and dipole–dipole interactions within the gel networks. Additionally, significant changes in the cholesterol proton signals were observed, suggesting the involvement of van der Waals forces in the supramolecular gel formation, as presented in Figure S3 (SI). The broadening and reduced intensity of the ^1^H NMR resonance peaks at lower temperatures can be attributed to restricted freedom of movement, such as the behavior in the gel state.^55^ As a result, the supramolecular gel structure was likely formed and stabilized by a combination of hydrogen bonding, dipole–dipole interactions, and van der Waals forces.

Furthermore, concentration-dependent ^1^H NMR was taken to obtain more insights into the noncovalent interactions influencing the development of self-assembled aggregated gels. When the gelator *Z*-**D** concentration was increased from 1.5 to 10.5 mM in a benzene-*d*_6_ solvent, a downfield shift in the −NH proton (H_j_) and triazole proton (H_h_) signals was observed. These shifts suggest the involvement of intermolecular hydrogen bonding and dipole–dipole interactions, as presented in Figures 3b and S4 (SI). This result is highly consistent with the temperature-dependent ^1^H NMR spectra. On the other hand, the proton signals of the stiff-stilbene aromatic ring did not exhibit significant chemical shift changes with increasing temperature and concentration, indicating that they did not engage in π–π stacking interactions.

**Figure 4:**
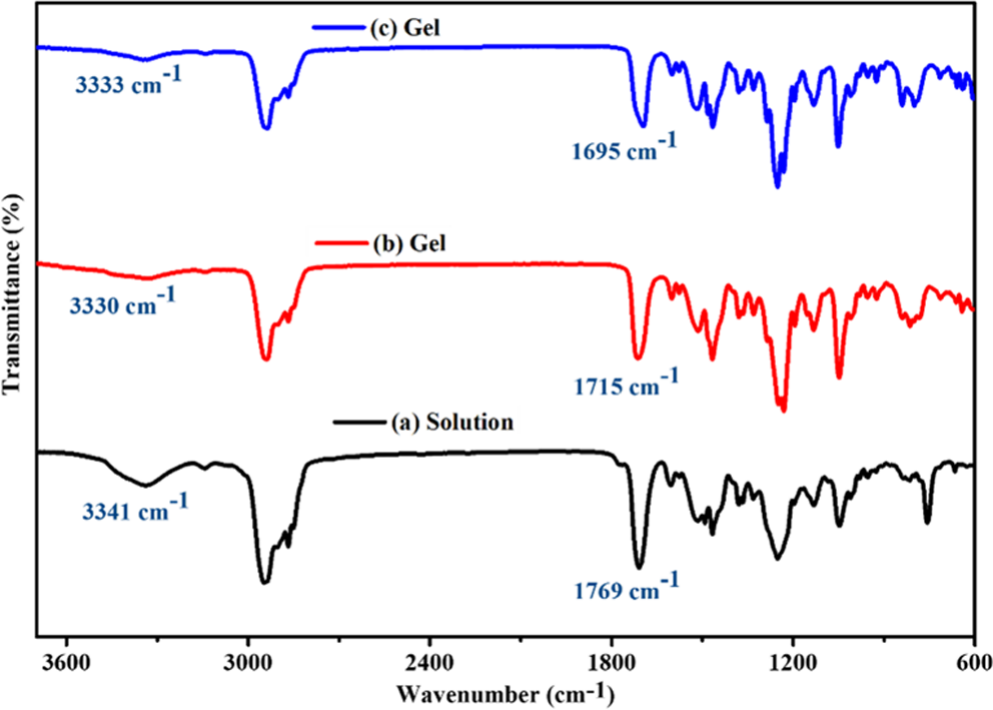
FT-IR spectra of the *Z*-**D** gelator in (a) CHCl_3_ solution, (b) ACN/DCM (v/v = 1:1) xerogel, and (c) toluene xerogel.

Besides temperature and concentration-dependent ^1^H NMR measurements, FT-IR measurement was also performed to investigate the existence and participation of hydrogen bonding for gelator (*Z*-**D**) self-aggregation. As presented in Figure 4a–c and Table S2 (SI) the FT-IR spectra of the gelator *Z*-**D** were examined in ACN/DCM (v/v = 1:1) and toluene gels, as well as in CHCl_3_ solution as a control system.

Chloroform (CHCl_3_) was found to be a good solvent for the *Z*-**D** gelator, causing the gelator molecule to exist as isolated individual components rather than in a cross-linked chain form. In the solution state, the gelator exhibited characteristic peaks at 3341 and 1769 cm^–1^ corresponding to N–H stretching and C=O stretching vibrations of the carbamate functionalities, respectively, as shown in Figure 4a. However, in the xerogel states formed in ACN/DCM (v/v = 1:1), the N–H stretching and C=O stretching vibrations were both shifted to lower wavenumbers of 3330 and 1715 cm^–1^, respectively, as shown in Figure 4b. Similarly, the typical bands corresponding to the stretching of carbamate N–H and C=O stretching vibrations for the toluene xerogels are observed at 3333 and 1695 cm^–1^, respectively, as presented in Figure 4c. The shift to lower wavenumbers and the decrease in peak intensities with increasing broadness indicate that the N–H protons are involved in hydrogen bonding interactions within the self-assembled gel network. The result provides additional insight into the self-assembly process of *Z*-**D**, indicating that hydrogen bonding interactions play a crucial role in stabilizing the gelator architecture in the presence of organic solvents.

Furthermore, to obtain additional evidence for the supramolecular self-assemblies of the *Z*-**D** gelator, variable temperature UV–vis absorption experiments were conducted. The absorption spectra of *Z*-**D** in toluene (20 μM) demonstrated the transition from the self-assembled species to the isolated individual molecular species as the temperature increased from 298 to 358 K, as shown in Figure S5 (SI). In the UV–vis spectra, a rise in the intensity of the absorption peak was noted with decreasing temperature. These results suggest the presence of self-assembled entities at room temperature, while molecularly dissolved individual species predominate at higher temperatures.^56^ The results from temperature-dependent UV–vis absorption experiments support the findings obtained from both ^1^H NMR and FT-IR spectroscopies. These combined results offer a more comprehensive understanding of the self-assembly processes and elucidate the nature of the interactions stabilizing the *Z*-**D** gels.

Based on the spectral changes observed in the temperature- and concentration-dependent ^1^H NMR experiments, as well as the temperature-dependent UV–vis absorption data, we conclude that in addition to van der Waals and dipole–dipole interactions, hydrogen bonding interactions between the –NH groups of the carbamate play a crucial role in the self-assembly processes of the *Z*-**D** gelator.

## Morphologies of Gels and Their Light-Induced Transitions

**Figure 5:**
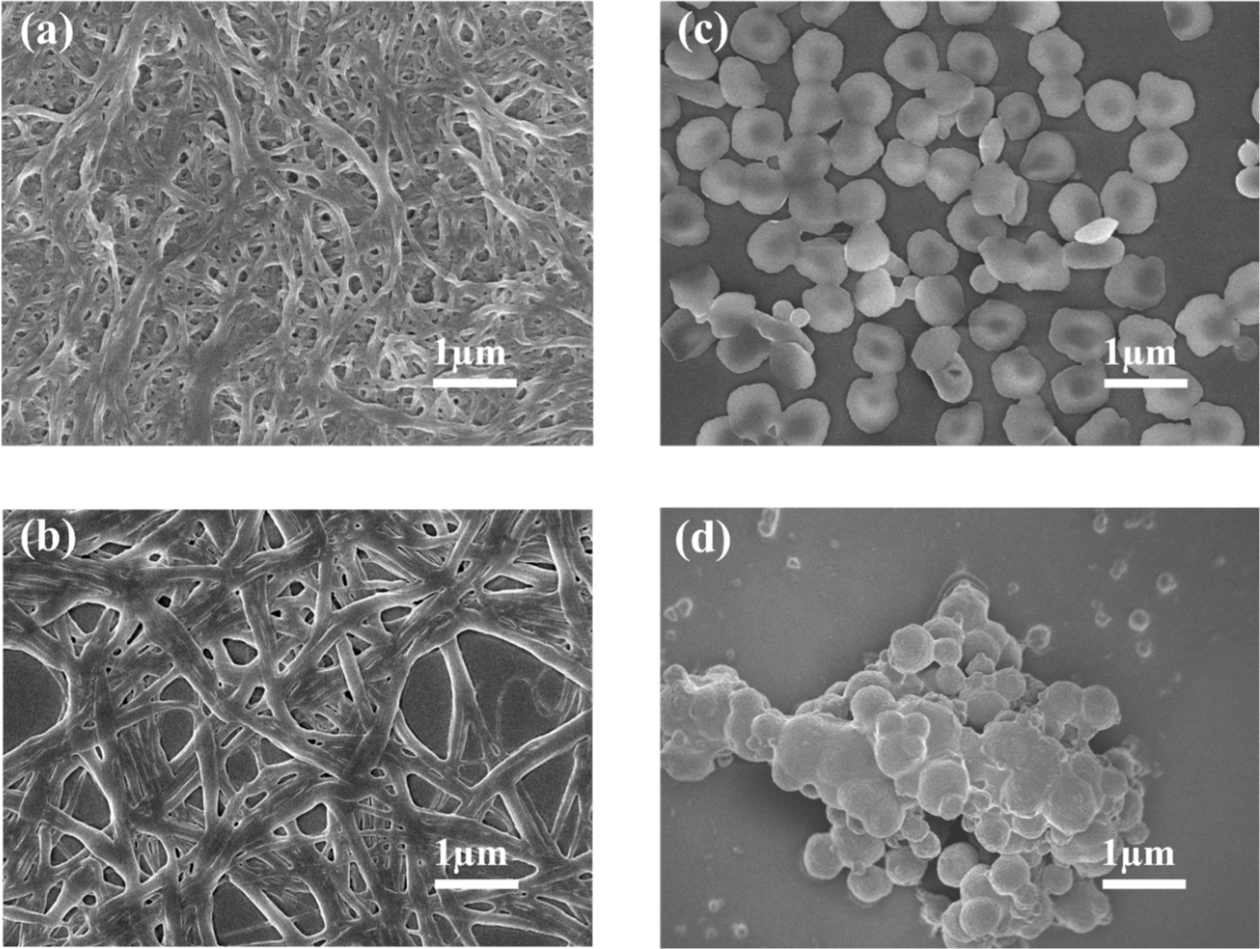
SEM images of *Z*-**D** gels and their light-triggered morphological transitions at the PSSs: (a) In ACN/DCM (v/v = 1:1) before and (c) after 385 nm UV light irradiation. (b) In toluene before and (d) after 385 nm UV light irradiation.

The morphologies of high-vacuum-dried self-aggregated xerogels at their minimum gelation concentrations (MGCs) were characterized by using field-emission scanning electron microscopy (FE-SEM). The SEM analysis offers significant insight into the microstructure and self-assembly behavior of the gelator molecules. The results provide strong evidence that the gelator molecule has a remarkable tendency for self-assembly, forming a nanofibrillar network structure across various gel-forming solvents as displayed in Figures 5a,b and S6a–e (SI). The fibrillar morphologies of *Z*-**D** xerogels observed in different solvents are not identical, suggesting that the microstructures of the gels are dependent on the nature of the solvents used for gelation. The formation of such nanofibrillar networks is driven by noncovalent interactions, including hydrogen bonding, dipole–dipole interactions, and van der Waals forces. These interactions facilitate the alignment and self-aggregation of individual gelator molecules into well-defined fibrils. The supramolecular gel, formed through noncovalent interactions, provides a means to realize macroscopic changes induced by light.^7,57^ Installation of a photosensitive stiff-stilbene unit into the bis-cholesterol derivatives of LMOG allows for further manipulation of its morphological changes and properties triggered by light. Since the most stable and strong gels were formed in ACN/DCM (v/v = 1:1) and toluene from mixed and single solvent categories, respectively, further SEM analysis to gain visual insights into light-induced morphological changes in gelator aggregation was carried out in these solvents. The MGCs of *Z*-**D** in toluene and ACN/DCM (v/v = 1:1) solvents were exposed to 385 nm light at room temperature for the duration required to reach the photostationary state. The corresponding gel-to-precipitate phase transitions of the ACN/DCM (v/v = 1:1) gel upon 385 nm UV light irradiation are demonstrated in Figure S2c.

**Scheme 3:**
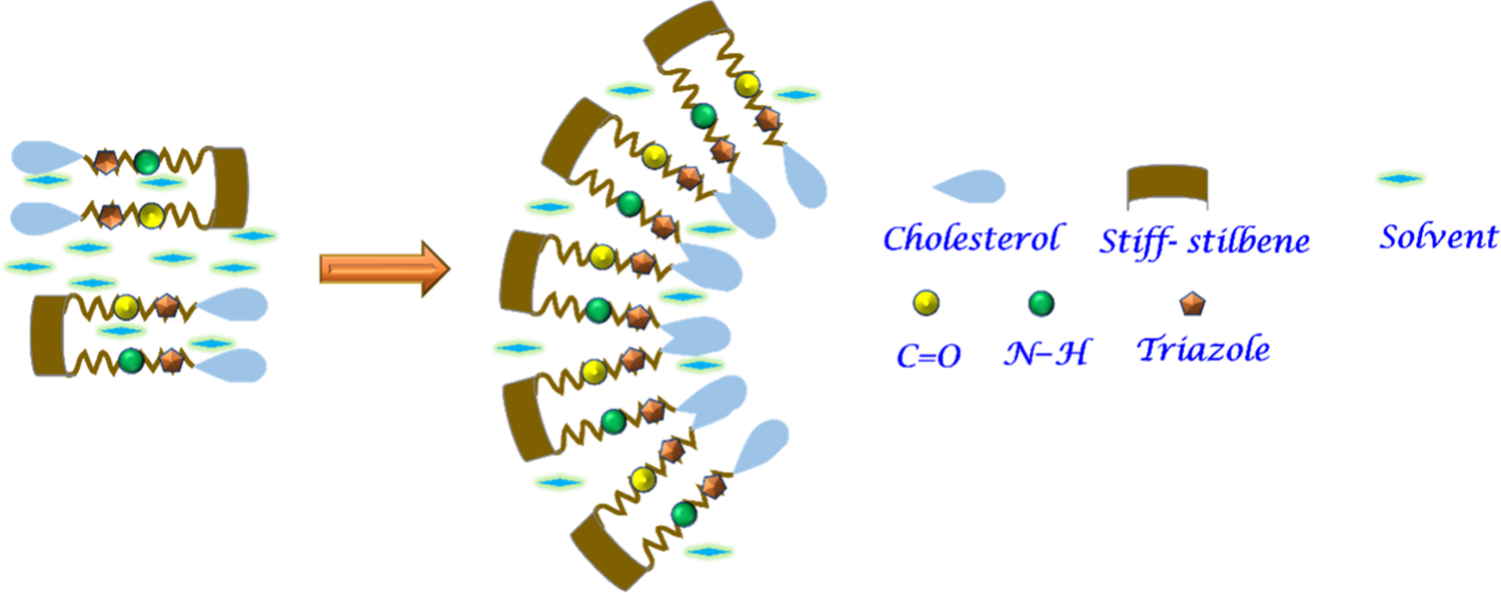
Schematic Illustration of the Proposed Molecular Packing Model of *Z*-**D** Gel

Upon irradiation, the *Z*-**D** gel underwent macroscopic collapse, transforming to a precipitate suspension. SEM analysis of the resulting precipitate solution revealed the disruption of the cross-linked fibrillar network, leading to the formation of irregular spherical aggregates, as evidenced by Figure 5c,d. This transformation from a fibrous network to irregular spherical aggregates suggests that the UV light induces the disruption of the fiber network and subsequent aggregation into nonregular spherical forms. However, the resulting precipitate does not reverse to the original gel state under 350 nm of irradiation. This is because the reverse process did not fully restore the original spectrum, only 39% of the *Z-*isomer being restored, as demonstrated by the UV–vis and ^1^H NMR reverse photoisomerization results presented in Figures 1a,b, and 2, respectively. Based on the results discussed above, the expected self-aggregation mode of the *Z*-**D** isomer in the gel state was proposed and schematically represented in Scheme 3. Initially, one-dimensional supramolecular structures of *Z*-**D** are driven by the van der Waals interactions between the cholesteryl units. These structures are further stabilized by H-bonding between the N–H and C=O groups of the carbamate units, leading to the self-assembly of fibrous structures.

## Rheological Studies

**Figure 6:**
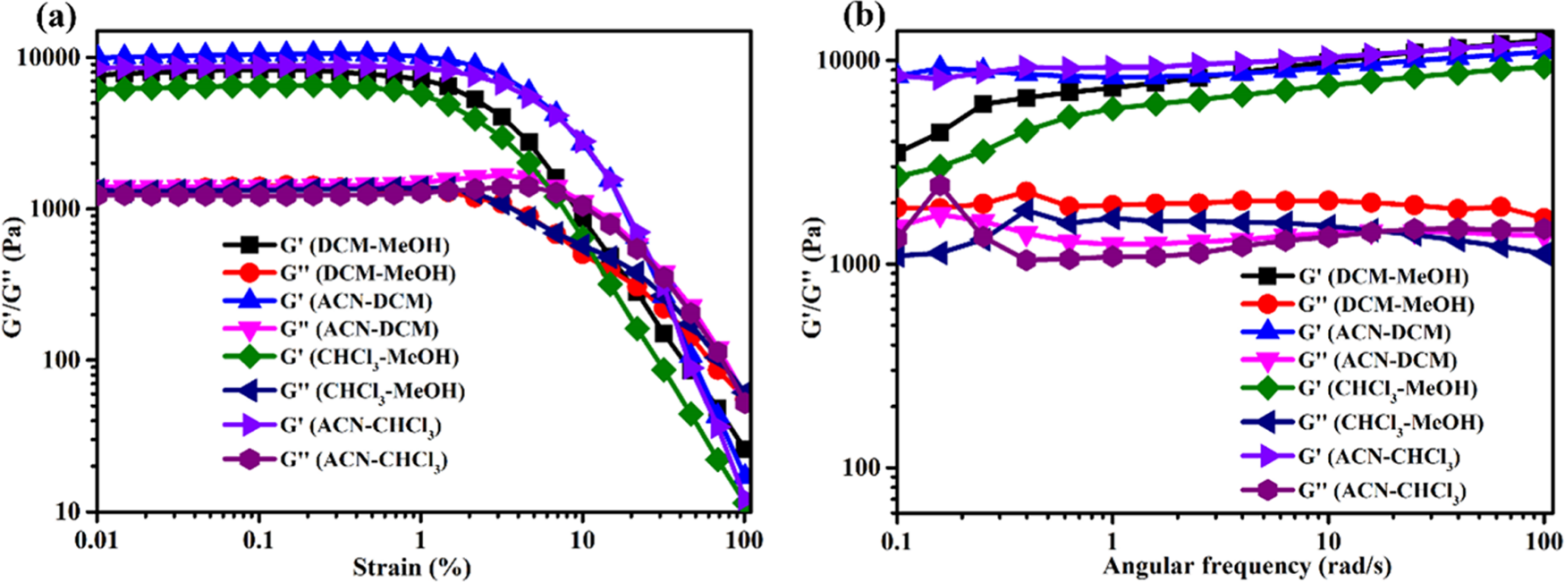
Storage modulus (*G*′) and loss modulus (*G*″) of the *Z*-**D** gel (MGC) in various mixed solvents as a function of (a) strain sweep and (b) frequency sweep at the strain of 0.03%.

The mechanical properties of the *Z*-**D** gels formed in both mixed and single solvents at their MGC values were investigated by using rheological methods. The amplitude sweep experiment was performed within the range of 0.01–100% shear strain at a constant angular frequency of 10 rad/s. The strain sweep and frequency experiments exhibited that the storage modulus (*G*′) of all gels was much higher than those of the loss modulus (*G*″), indicating the characteristics of true gels (see Figures 6 and S7 (SI)). The higher storage modulus (*G*′) compared to the loss modulus (*G*″) in all the tested gels suggests that the gels are well-formed and possess strong structural integrity. The *G*′ of each gel is found to be larger than the corresponding *G*″ at low-stress values, indicating the predominately elastic nature of the gels.^58^ In all solvents of *Z*-**D** gels, above the critical shear stress, the value of *G*″ exceeds that of *G*′, with sharp decreases observed in both values across all systems, as clearly shown in Figures 6a and S7a (SI). This indicates partial disruption of the 3D gel networks.

Frequency sweep is the most useful technique for determining the performances of material tolerance to external forces.^59^ Accordingly, the values of *G*′ and *G*″ for all gelating solvents were measured as a function of angular frequency from 0.1 to 100 rad/s under a constant shear stress of 0.03%, as depicted in Figures 6b and S7b (SI). The *G*′ of both mixed and single solvent gels consistently remains higher than *G*″ as the angular frequency gradually increases from 0.1 to 100 rad/s, indicating that the system behaves as a true gel.^60^ However, the values of *G*′ and *G*″ were not entirely constant across the entire frequency range. This variation might be attributed to the high volatility nature of the organic solvents, which can affect the viscoelastic properties of the gel systems.

## Conclusions

A novel *Z-*isomer of stiff-stilbene bridged bis-cholesterol (*Z*-**D**) was designed and synthesized, which can be converted into its *E*-isomer (*E*-**D**) with 100% conversion upon exposure to 385 nm UV light. *Z*-**D** was found to be supergelators in a mixed solvent of ACN/DCM (v/v = 1:1), DCM/MeOH (v/v = 1:2), ACN/CHCl_3_ (v/v = 2:1), and CHCl_3_/MeOH (v/v = 1:2), with MGCs as low as 0.2% w/v. These gels formed rapidly without the support of any external forces upon the addition of antisolvents into the vial containing the gelator and its dissolving solvents. The gelator molecule *Z*-**D** entraps organic solvents and organizes itself into 3D fibrillar networks in various single and mixed solvents, as confirmed by SEM analysis. Temperature- and concentration-dependent ^1^H NMR spectroscopy, temperature-dependent UV-absorption, and FT-IR measurements revealed that the *Z*-**D** gelator engages in various noncovalent interactions, including van der Waals forces, hydrogen bonding, and dipole–dipole interactions, to facilitate the formation of the gel network. Upon irradiation with 385 nm light, the gel networks disintegrated into a precipitate suspension, resulting in the transformation of the fibrous structures into irregular spherical aggregates. This breakdown is due to the conformational change in bis-cholesterol **D** from the *Z* to the *E* form, resulting in the formation of a precipitate suspension. Furthermore, rheological analysis revealed that the gels formed from a solvent mixture exhibited superior mechanical strength compared to gels formed from single nonpolar solvents. This work will expand the library of LMOGs and potentially pave the way for developing new functional materials, particularly stimuli-responsive bis-cholesterol materials with tunable properties.

## Experimental Section

### General Experimental Method

All chemicals were purchased from Sigma-Aldrich and used without further purification, unless otherwise noted. All reactions were conducted under a nitrogen atmosphere to ensure an inert environment. The *Z*–*E* photoisomerization of bis-cholesterol **D** was carried out at ambient conditions, with the sample cell positioned 5 cm from the UV light source (LED lamp) to maintain consistent irradiation conditions. Detailed experimental methods are provided in the Experimental Section of the Supporting Information.

### Synthesis Procedure

The detailed synthesis protocols for preparing known compounds are depicted in the Supporting Information (see Schemes S1 and S2).

#### Synthesis of Gelator *Z*-**D**

Copper(II) sulfate pentahydrate (CuSO_4_·5H_2_O, 0.33 g, 1.34 mmol) and sodium ascorbate (1.3 g, 6.68 mmol) were added to a mixed solution of compound **8** (3.1 g, 6.68 mmol) and compound **6** (1.28 g, 3.18 mmol) in a solvent mixture of THF and water (15:3, v/v, 18 mL). The reaction mixture was stirred at 60 °C for 48 h. After the mixture was cooled to room temperature, THF was removed under reduced pressure. The remaining residue was extracted with DCM, and the organic layer was washed sequentially with water and brine solutions. The organic phase was dried over anhydrous Na_2_SO_4_, concentrated, and purified by column chromatography on silica gel using a mixture of DCM and methanol (20:1, v/v) as the eluent. This process yielded compound *Z*-**D** as a light-yellow solid (2.79 g, 65% yield). ^1^H NMR (400 MHz, CDCl_3_) δ 7.83 (s, 1H), 7.50 (d, *J* = 2.5 Hz, 2H), 7.17 (d, *J* = 8.3 Hz, 2H), 6.71 (dd, *J* = 8.2, 2.3 Hz, 2H), 5.59 (s, 2H), 5.44–5.25 (m, 2H), 4.75 (s, 4H), 4.48–4.38 (m, 6H), 4.33–4.18 (m, 4H), 2.94–2.83 (m, 4H), 2.89–2.70 (m, 4H), 2.39–0.35 (m, 86H). ^13^C{^1^H} NMR (101 MHz, CDCl_3_) δ 156.4, 141.9, 141.8, 139.1, 135.6, 126.1, 122.6, 115.0, 108.1, 74.8, 66.9, 56.9, 56.3, 50.1, 42.5, 39.9, 39.7, 38.7, 37.1, 36.7, 36.3, 35.1, 35.5, 32.0, 32.0, 30.0, 28.4, 28.3, 28.2, 24.4, 24.0, 22.1, 22.7, 21.2, 19.5, 18.9, 12.0. HRMS (ESI) *m*/*z*: [M + H]^+^ Calcd for C_84_H_121_N_8_O_6_ 1337.9404; found 1337.9429.

#### Synthesis of *E*-**D** Isomer

To synthesize the *E*-**D** isomer, a degassed solution of *Z*-**D** (30 μM) in CH_2_Cl_2_ was irradiated with light of 385 nm wavelength for 50 s. After irradiation, the conversion to the *E*-**D** isomer was nearly quantitative with an observed efficiency of approximately 100%. The successful isomerization was confirmed by ^1^H NMR spectroscopy, which showed significant shifts in the proton signals of the aromatic stiff-stilbene rings, confirming the formation of the *E*-**D** isomer. ^1^H NMR (400 MHz, CDCl_3_) δ 7.76 (s, 2H), 7.20 (d, *J* = 8.3 Hz, 2H), 7.10 (d, *J* = 2.4 Hz, 2H), 6.73 (dd, *J* = 8.2, 2.3 Hz, 2H), 5.33 (s, 2H), 5.19 (s, 2H), 4.75 (t, *J* = 5.0 Hz, 4H), 4.56–4.40 (m, 6H), 4.38 (t, *J* = 5.0 Hz, 4H), 3.26–3.08 (m, 4H), 3.10–3.01 (m, 4H), 2.42–0.53 (m, 86H). ^13^C{^1^H} NMR (101 MHz, CDCl_3_) δ 156.8, 156.1, 145.3, 144.5, 140.5, 139.7, 135.8, 125.4, 123.2, 122.5, 113.5, 110.9, 74.6, 66.7, 56.6, 56.1, 49.9, 42.2, 39.7, 39.5, 38.5, 36.9, 36.5, 36.1, 35.8, 32.6, 31.8, 30.2, 29.7, 28.2, 28.1, 28.0, 24.2, 23.8, 22.8, 22.5, 20.96, 19.2, 18.7, 11.8. HRFD-MS: *m*/*z*: Calcd for C_84_H_120_N_8_O_6_ 1336.9336; found 1336.9348.

## Data Availability

The data underlying this study are available in the published article and its Supporting Information.

## Supporting Information Available

Detailed experimental methods, synthetic route of all compounds, and their characterizations using NMR, 2D COSY, FT-IR, and HRMS spectroscopic techniques; UV–vis absorption and fluorescence spectra, and FE-SEM images (PDF)
