# Supplementary material for: Stiff-Stilbene-Linked Bis-Cholesterol: Synthesis and Investigation of Its Supramolecular Gelation and Photophysical Behaviors
Source: ACS Omega. 2024 Dec 26;10(1):1789–99. doi: 10.1021/acsomega.4c10136 (PMC11740128; doi:10.1021/acsomega.4c10136)
Supplement: Supplementary file 1 — ao4c10136_si_001.pdf [file ao4c10136_si_001.pdf]

Supporting Information

Stiff-Stilbene-Linked Bis-Cholesterol: Synthesis and Investigation of Its Supramolecular Gelation and Photophysical Behaviors

Dagninet Yeshiwas Alene [a] *and Wen-Sheng Chung* [*] [a]

[a] Department of Applied Chemistry, National Yang Ming Chiao Tung University, Hsinchu 30050, Taiwan, ROC.

[*]Corresponding Author:

Prof. Wen-Sheng Chung

Department of Applied Chemistry

National Yang Ming Chiao Tung University, Hsinchu, Taiwan, ROC

Email: wschung@nycu.edu.tw

Table of Contents

Experimental Section S2–S3

Synthesis of compound **6**; synthesis and characterization of compounds **7** and **8** S4–S5

Supporting figures and images S6–S12

^{1}H NMR, ^{13}C NMR, and HRMS spectra of new compounds S13–S19

References S20

Experimental Section

Materials, Instrumentation, and Methods. The compounds were purified after each reaction using silica gel column chromatography. Thin-layer chromatography (TLC) analyses were conducted on aluminum-backed plates, with ultraviolet (UV) light used for spot visualization. All ^{1}H and ^{13}C NMR experiments, except for temperature-dependent ones, were recorded at room temperature using Agilent NMR400-VNMRs and JEOL NMR400 spectrometers, with $CDCl_3$ as the solvent. Chemical shifts are reported in parts per million (ppm). Temperature-dependent ^{1}H NMR experiments were performed using a Varian-NMR600-Inovas spectrometer. Solvent chemical shifts were calibrated to the solvent residue reference peak, for ^{1}H NMR ($CDCl_3$ = 7.26 ppm), and ^{13}C NMR ($CDCl_3$ = 77.16 ppm). Coupling constants (*J*) are expressed in hertz (Hz). High-resolution mass spectrometry-electrospray ionization (HRMS-ESI) and High-resolution field desorption (HRFD) measurements were performed using an HD Q-TOF mass spectrometer (Bruker, Germany), and JEOL JMS-T200GC, respectively. UV-vis absorption and fluorescence spectra were recorded at ambient temperature using an HP-8453 spectrometer and a HORIBA FluoroMax-4 spectrometer, respectively, with a 1 cm quartz cell used for both measurements. The photoswitching study of *cis* to *trans* and *trans* to *cis* isomerization was carried out by using a 385 nm LED lamp (HJS-480-0-24) and a Rayonet RPR-100 photochemical reactor equipped with 350 nm lamps (450 W), respectively. The reported morphologies were investigated using a field-emission scanning electron microscope (FE-SEM, JEOL JSM-7401). Samples were prepared using the drop-casting technique, where a small amount of gel was manually cast onto glass slices attached to copper and carbon tapes, and then the solvent was slowly removed under a high vacuum overnight. A thin layer of Pt was deposited via sputtering onto the samples before SEM examination to minimize sample charging. The rheological measurements of supramolecular gels were performed on the MCR 302 rheometer (Anton Paar, Austria) using a 25 mm diameter

sandblast parallel plate geometry to obtain the storage modulus (G′) and loss modulus (G″) of the supramolecular gels. FTIR spectroscopy data were recorded using a Bruker IR spectrophotometer (Vertex 80v and Tensor 27 models). All reactions requiring heat were conducted using a heating mantle as the heat source.

Gelation tests: The gelation properties of the *Z*-**D** compound were characterized in various organic solvents by the "stable to inversion in a test tube" method. In a single solvent such as toluene, benzene, and *p*-xylene gelation process, first gelator and solvents were put into a sealed vial, and heated using a heat gun until the solid was completely dissolved. Then the gels formed as the hot solutions cooled to room temperature, which was confirmed by observing a stable gel when the vial was inverted. Whereas in a mixed solvent such as ACN/DCM (v/v = 1:1), DCM/MeOH (v/v = 1:2), ACN/$CHCl_3$ (v/v = 2:1), and $CHCl_3$/MeOH (v/v = 1:2), the gelator was first weighed and placed in a vial. Subsequently, a solvent capable of dissolving the gelator was added, resulting in a homogeneous solution. Upon the addition of the appropriate ratio of the anti-solvents, the solution rapidly transformed into a gel without the need for any mechanical agitation. The minimum gelation concentration (MGC) was determined by incrementally adding 100 μL of appropriate solvents to a vial containing a precisely weighed amount of the gelator (*Z*-**D**).

Synthesis and Characterizations of Compounds

Scheme S1. Synthesis of compound **6**

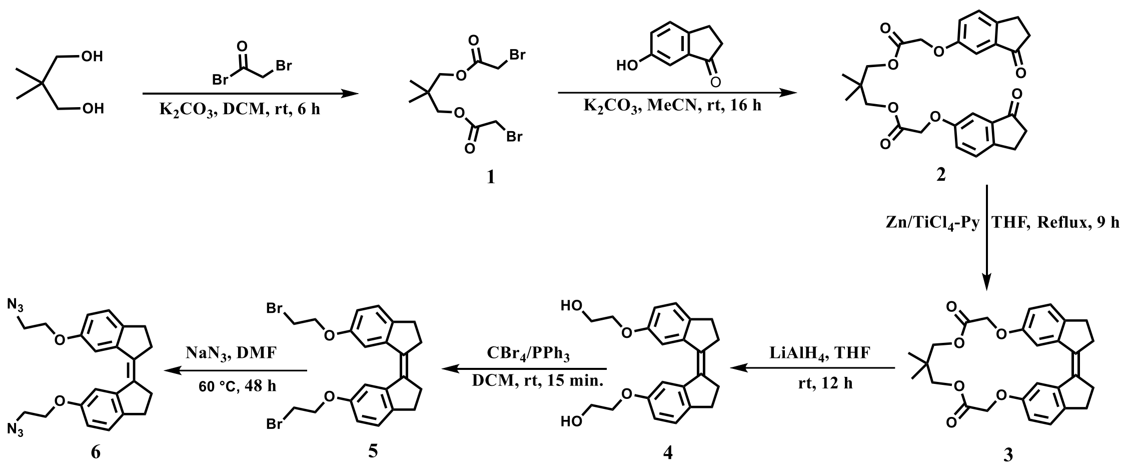

Compounds **1–6** were synthesized based on our previously reported procedures and the procedures described in the other literature. [S1-S2]

Scheme S2. Synthesis of compound **8**

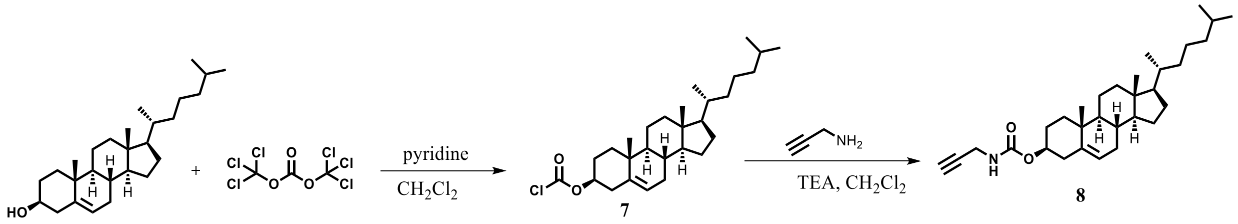

We synthesized compound **8** based on procedures as described in the literature.[S3]

Preparation of Cholesterol Chloroformate (7). Cholesterol (3.38 g, 8.74 mmol) was dissolved in 30 mL of anhydrous dichloromethane (DCM) and stirred for 30 minutes at room temperature. Subsequently, triphosgene (0.86 g, 2.9 mmol) and a few drops of pyridine were added to the solution at 0 °C, using an ice bath to maintain the temperature. The reaction mixture was stirred

for 1 hour at 0 °C and then for an additional 3 hours at room temperature. Upon completion of the reaction, the solvent was evaporated under reduced pressure. The resulting residue was purified by column chromatography on silica gel (SiO_2) using dichloromethane as the eluent (DCM: 100). This purification process yielded the desired compound 8 as a white solid (3.36 g, 85% yield). ^{1}H NMR (400 MHz, $CDCl_3$) δ 5.42 (d, J = 5.1 Hz, 1H), 4.79–4.59 (m, 1H).

Preparation of Compound 8. A stirred solution of propargylamine (1.00 mL, 14.28 mmol) and triethylamine (TEA, 4.00 mL, 28.58 mmol) in dichloromethane (DCM, 80 mL) was prepared. To this solution, a solution of compound **7** (6.42 g, 14.28 mmol) in DCM (40 mL) was added dropwise over 3 hours at 0 °C. The reaction mixture was then stirred for an additional 5 h at room temperature. Following the completion of the reaction, the mixture was filtered. The filtrate was sequentially washed with 0.01 M hydrochloric acid (3 × 50 mL) and pure water (3 × 50 mL). The organic phase was dried over anhydrous sodium sulfate (Na_2SO_4). The crude product was subsequently purified by silica gel column chromatography using DCM as the eluent, yielding compound **8** as a white solid (3.31 g, 50% yield). ^{1}H NMR (400 MHz, $CDCl_3$) δ 5.37 (d, J = 5.4 Hz, 1H), 4.79 (s, 1H), 4.62–4.42 (m, 1H), 4.09–3.85 (m, 2H), 2.41–0.58 (m, 48H). ^{13}C{^{1}H} NMR (101 MHz, $CDCl_3$) δ 155.7, 139.8, 122.8, 80.0, 75.1, 71.6, 56.8, 56.2, 50.1, 42.4, 39.8, 39.6, 38.6, 37.1, 36.7, 36.3, 35.9, 32.0, 32.0, 30.8, 28.4, 28.2, 28.1, 24.4, 24.0, 23.0, 22.7, 21.2, 19.5, 18.8, 12.0. HRMS (ESI) *m/z*: $[M+H]^+$ Calcd for $C_{31}H_{50}NO_2$ 468.3836; Found 468.3838.

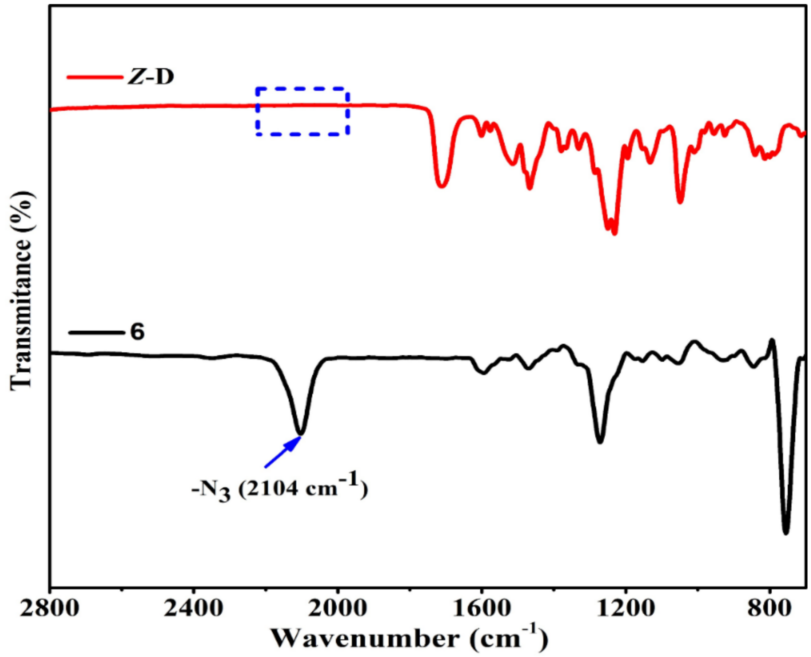

Figure S1. FT-IR spectra of compounds **6** and *Z*-**D**.

Percent of conversion for *E* to *Z* isomerization: The mixture of *Z* and *E* isomers in a 39:61 conversion ratio was calculated using the equation below. This calculation was based on the ^{1}H NMR integrals of peaks labeled as a-e, as depicted in Scheme 1 and Figure 2 of the manuscript.

$$\% \text{ Conversion} = \frac{Z\text{-}\mathbf{D}}{Z\text{-}\mathbf{D} + E\text{-}\mathbf{D}} \times 100$$

Where *Z*-**D** stands for H_a, H_b, H_c, H_d and H_e protons belong to *Z*-**D** at PSS_{350} and *E*-**D** represents those protons belonging to *E*-**D** at PSS_{350}.

Table S1. Gelation properties of *Z*-**D** and the critical gelation concentrations (w/v%) in various organic solvents.[a]

Solvents	States	
	Z-**D**	*E*-**D**
methanol	I	I
ethanol	I	I
n-butanol	I	I
n-propanol	P	P
isopropanol	P	P
DMF	I	I
DMSO	I	I
toluene	G (0.8)	P
p-xylene	G (1.5)	P
benzene	G (1.8)	P
dichloromethane	S	P
chloroform	S	S
dichloroethane	S	P
1,4-dioxane	S	P
cyclohexane	I	I
n-hexane	I	I
n-pentane	I	I
acetonitrile	I	I
THF	S	S

[a]Abbreviations: P = precipitates G = gel; numbers in brackets represent their MGC values in w/v% = [(g/100 mL) %].

Table S2. FT-IR data for the solution of *Z*-**D** gelator in $CHCl_3$ and its gels in ACN/DCM and toluene solvents

	λ_{max} (cm^{-1})	
	N–H stretch	C=O stretching vibrations
Z-**D** ($CHCl_3$) solution	3341	1769
Z-**D** (ACN/DCM) gel	3330	1715
Z-**D** (Toluene) gel	3333	1695

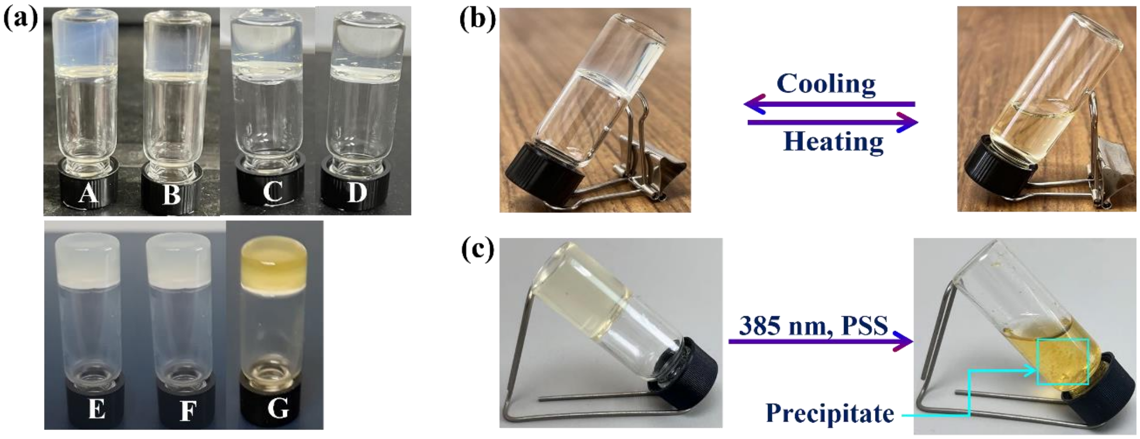

Figure S2. (a) photographs of *Z*-**D** gels that remain stable at room temperature in (A) ACN/DCM (v/v = 1:1), (B) DCM/MeOH (v/v = 1:2), (C) ACN/$CHCl_3$ (v/v = 2:1), and (D) $CHCl_3$/MeOH (v/v = 1:2), (E) toluene, (F) benzene, (G) *p*-xylene, (b) thermally-induced sol-gel transitions of the *Z*-**D** gel, and (c) light-induced gel-to-precipitate transition at the PSS.

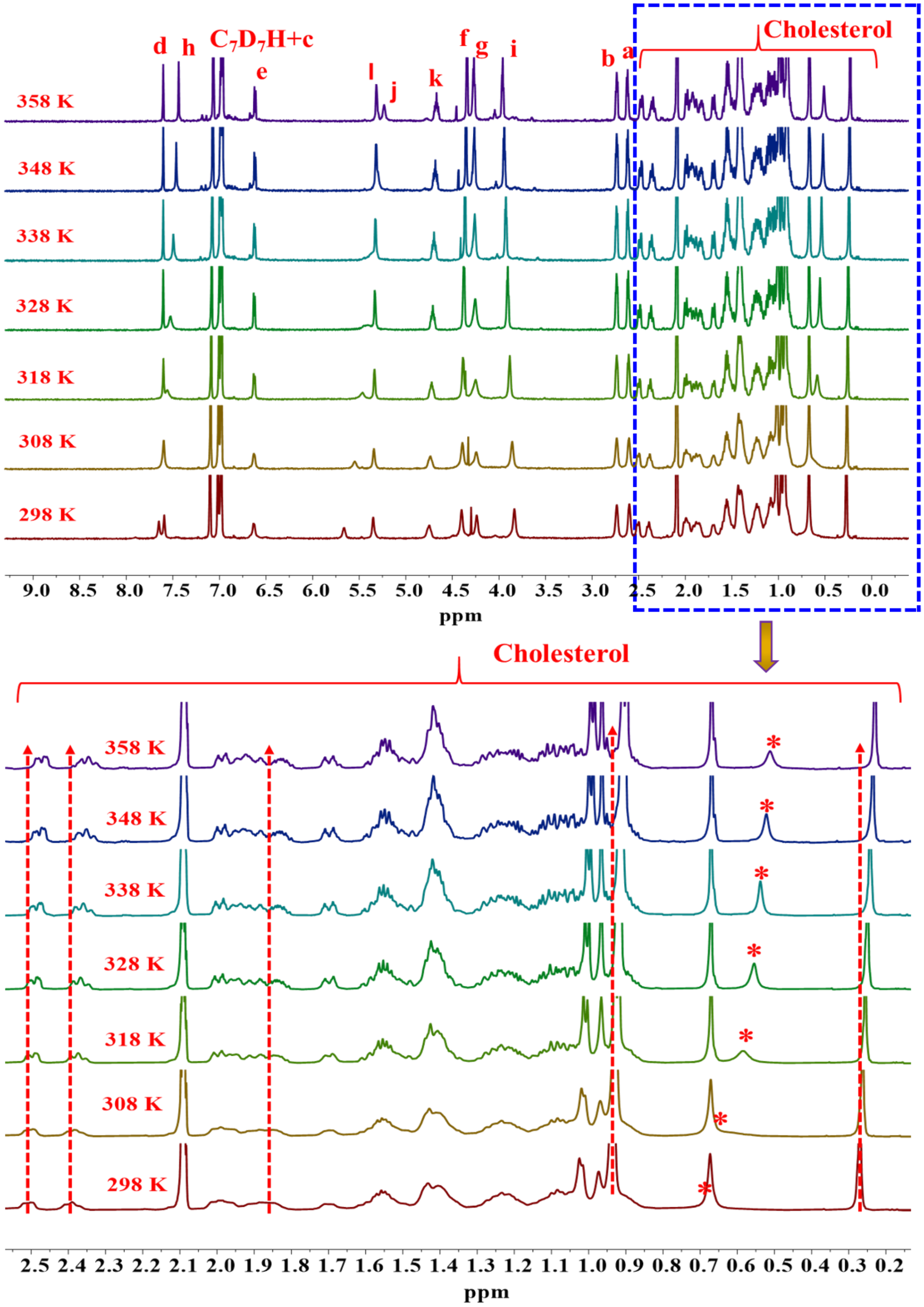

Figure S3. Temperature-dependent ^{1}H NMR spectra of *Z*-**D** in toluene-d_8 at a concentration of 1.5 (w/v%).

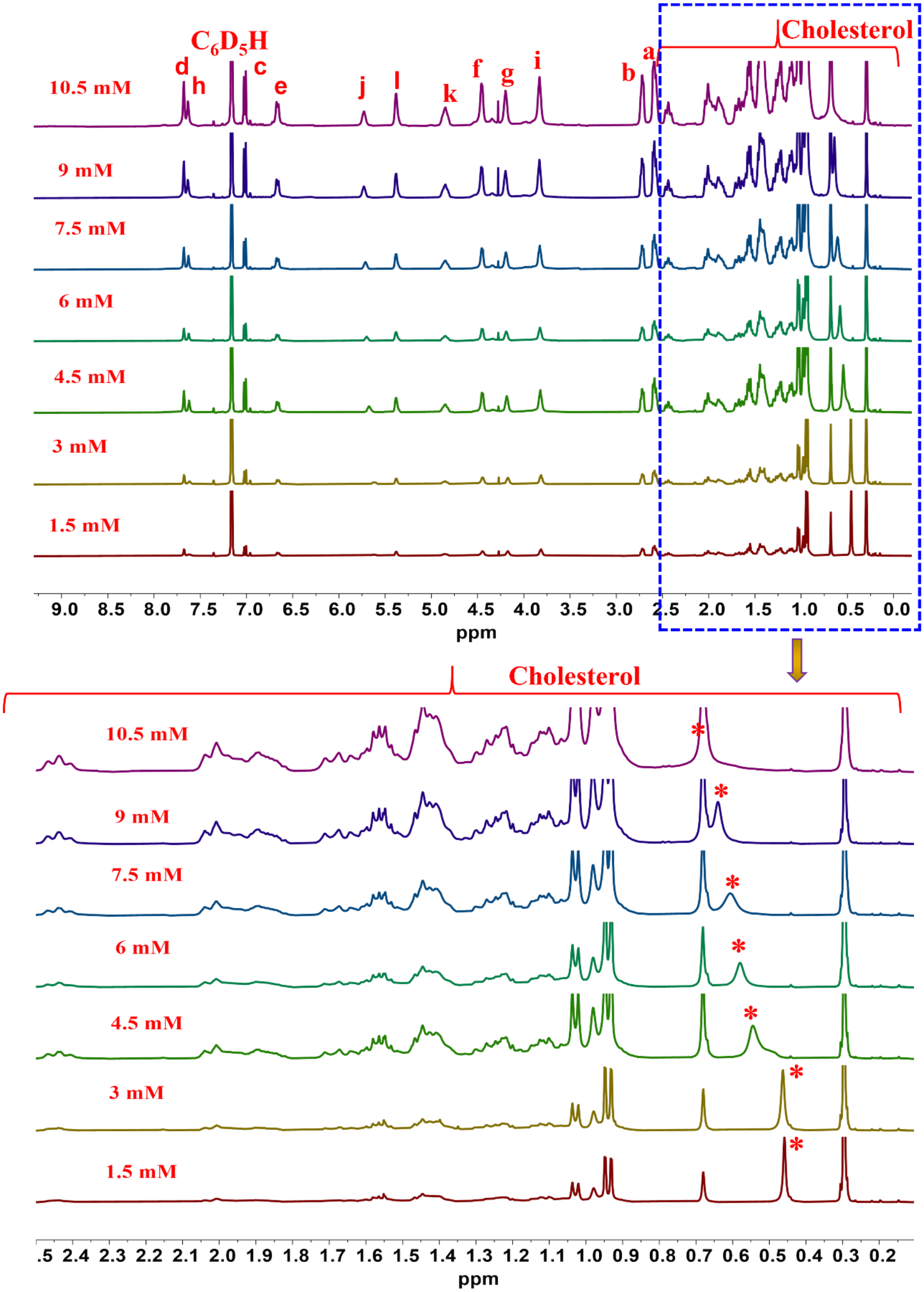

Figure S4. Concentration-dependent ^{1}H NMR spectra of *Z*-**D** in benzene-d_6.

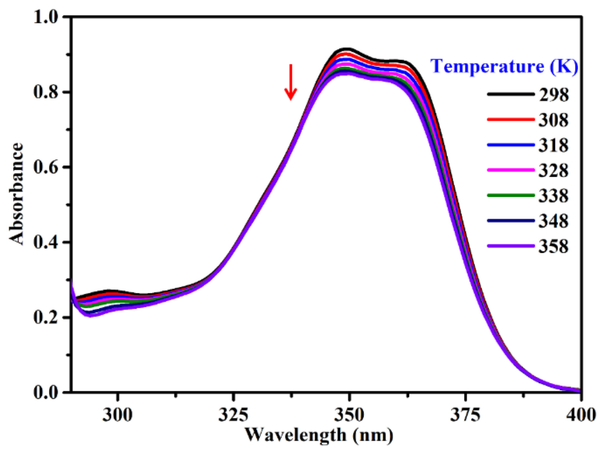

Figure S5. Changes in the UV–Vis absorption spectra of *Z*-**D** (20 μM) upon increasing temperature from 298 to 358 K.

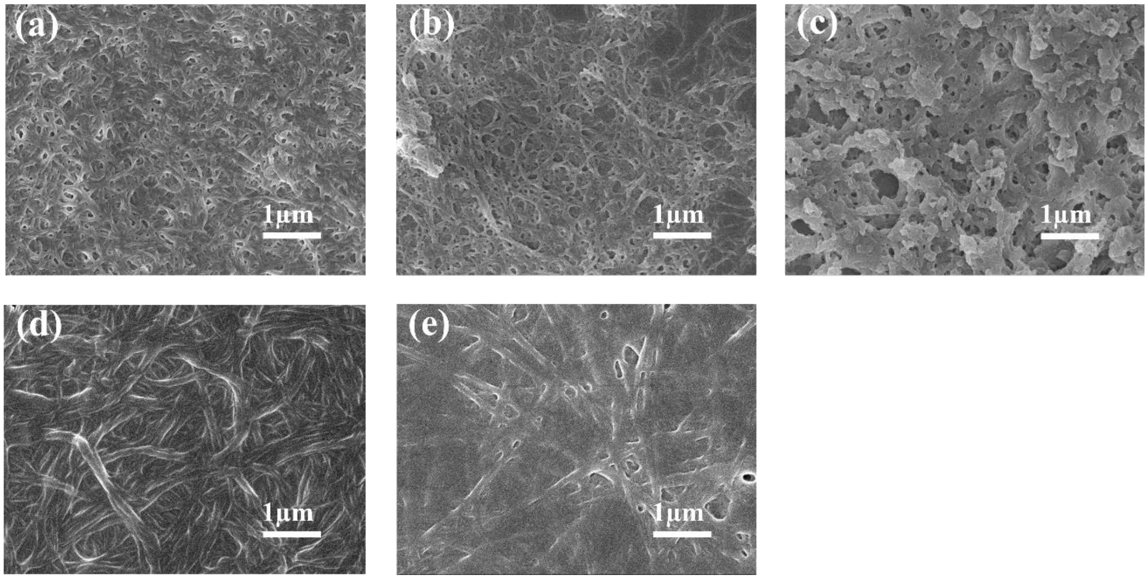

Figure S6. SEM images of *Z*-**D** gels at MGCs in (a) ACN/$CHCl_3$ (v/v = 2:1), (b) $CHCl_3$/MeOH (v/v = 1:2), (c) DCM/MeOH (v/v = 1:2), (d) benzene, and (e) *p*-xylene.

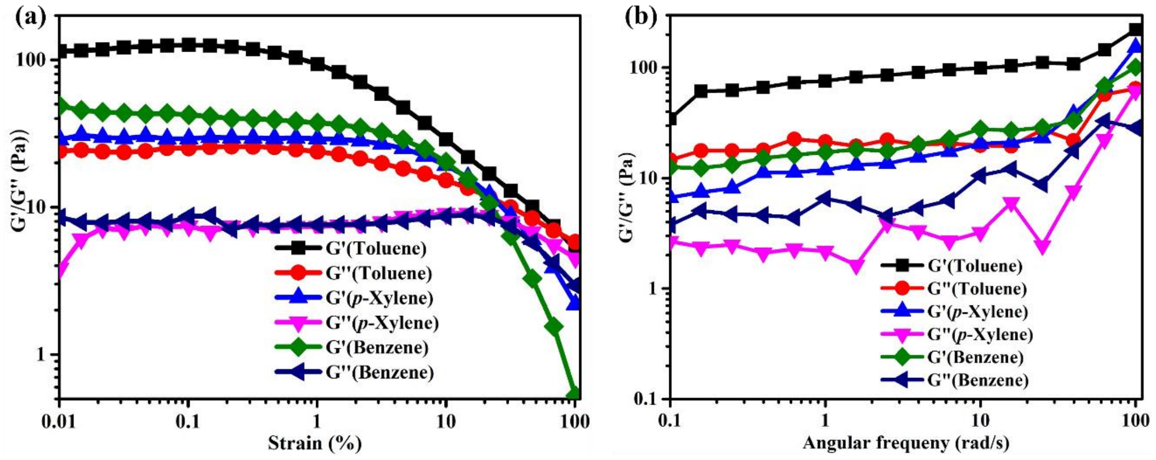

Figure S7. Storage modulus (G′) and loss modulus (G″) of the *Z*-**D** gel (MGC) in toluene, benzene, and *p*-xylene solvents as a function of (a) strain sweep, and (b) frequency sweep at the strain of 0.03%.

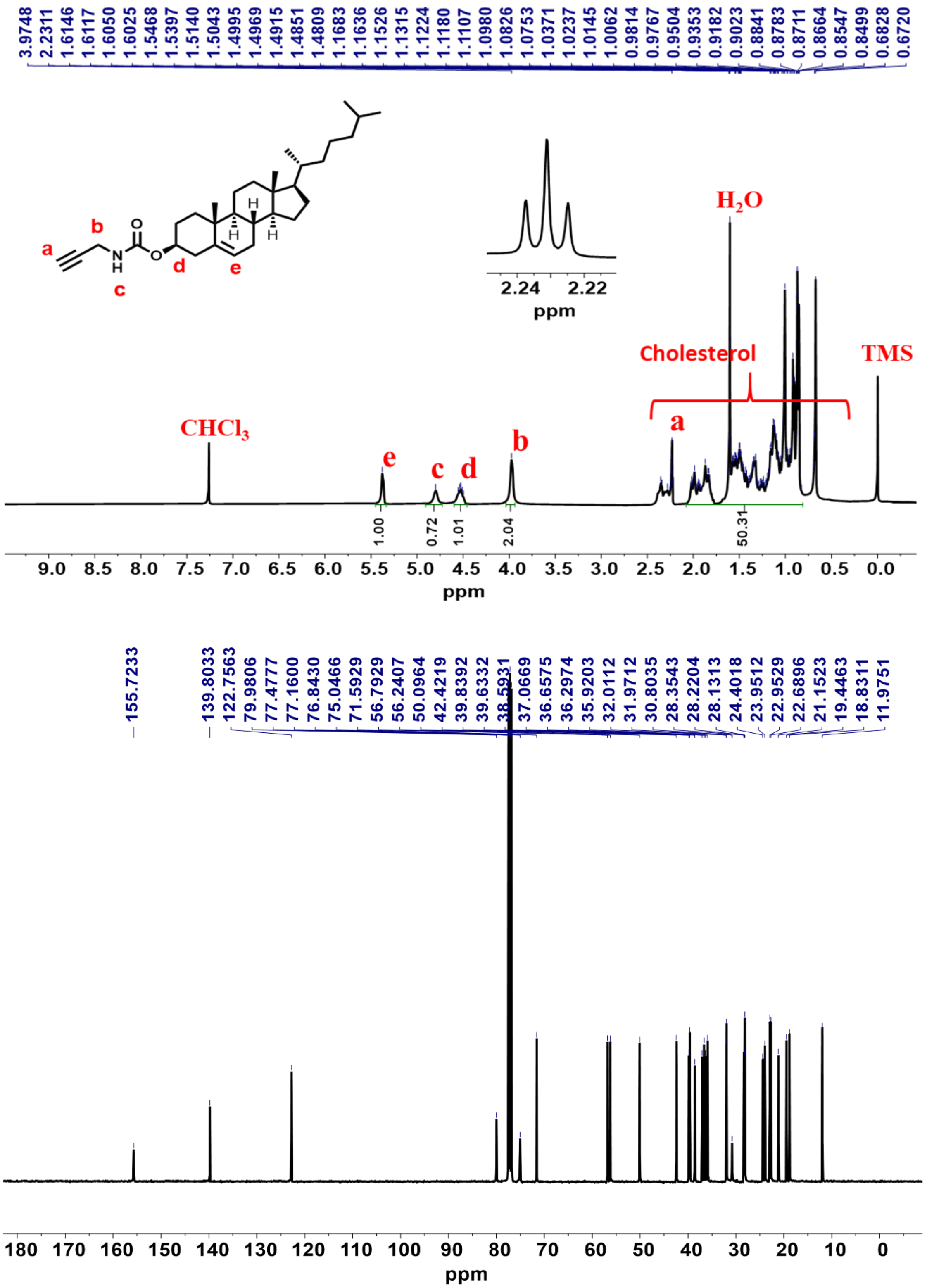

Figure S8. ^{1}H and ^{13}C NMR spectra of compound **8** in $CDCl_3$.

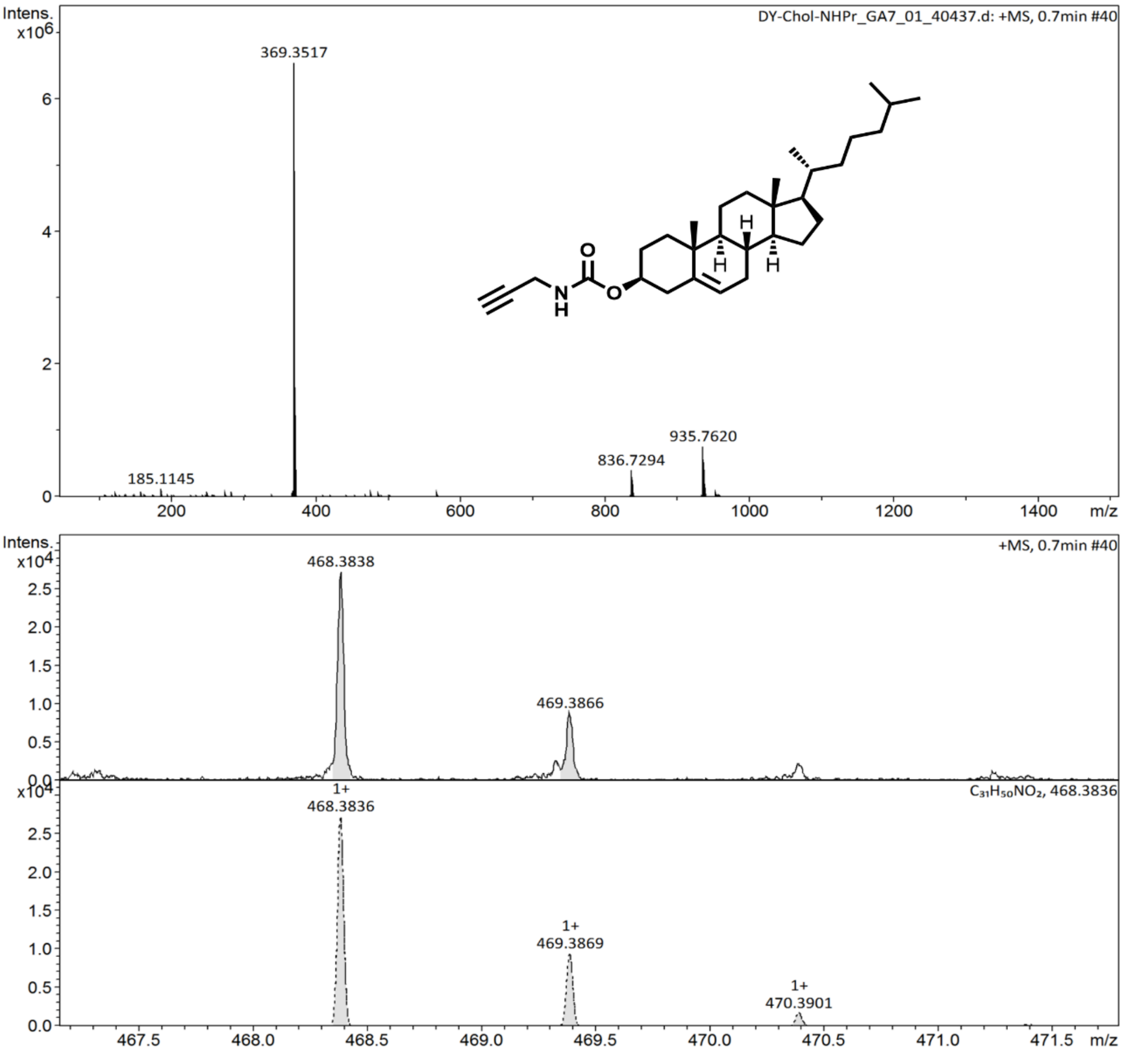

Meas. m/z	#	Ion Formula	m/z	err [ppm]	mSigma	# Sigma	Score	rdb	e⁻ Conf	N-Rule	Adduct
468.3838	1	C31H50NO2	468.3836	0.4	33.0	1	100.00	7.5	even	ok	M+H

Figure S9. HRMS-ESI data of compound **8**.

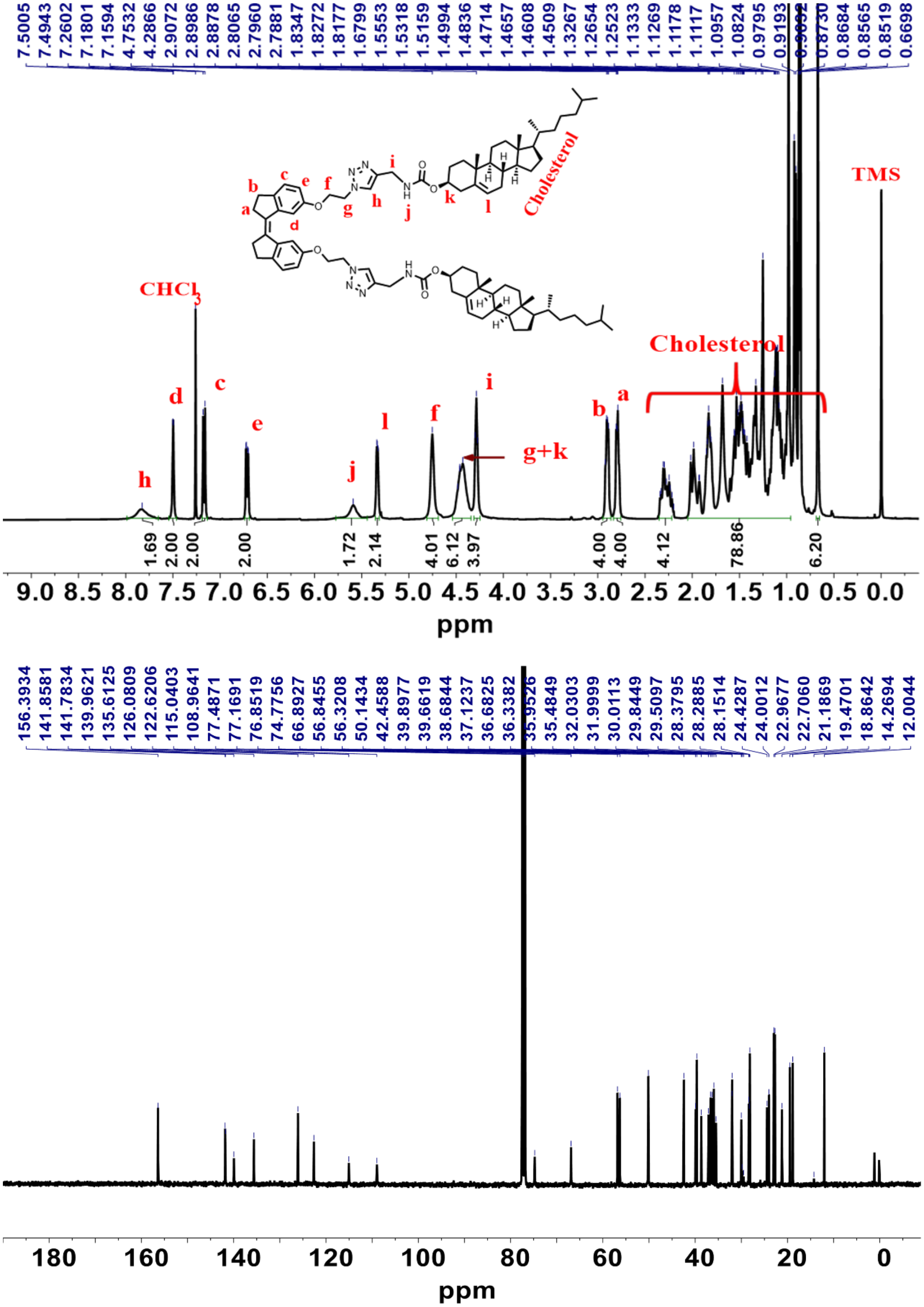

Figure S10. ^{1}H and ^{13}C NMR spectra of compound *Z*-**D** in $CDCl_3$.

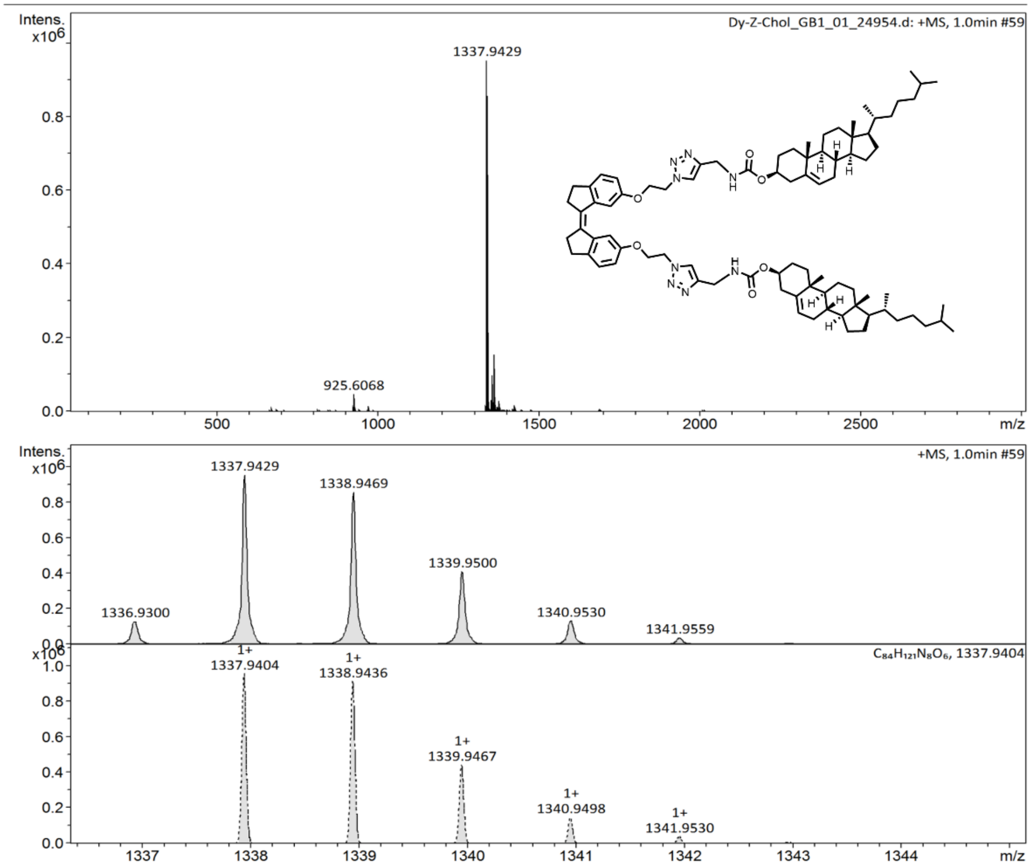

Meas. m/z	#	Ion Formula	m/z	err [ppm]	mSigma	# Sigma	Score	rdb	e⁻ Conf	N-Rule	Adduct
1337.9429	1	C84H121N8O6	1337.9404	-1.9	26.9	1	100.00	28.5	even	ok	M+H

Figure S11. HRMS-ESI data of compound *Z*-**D**.

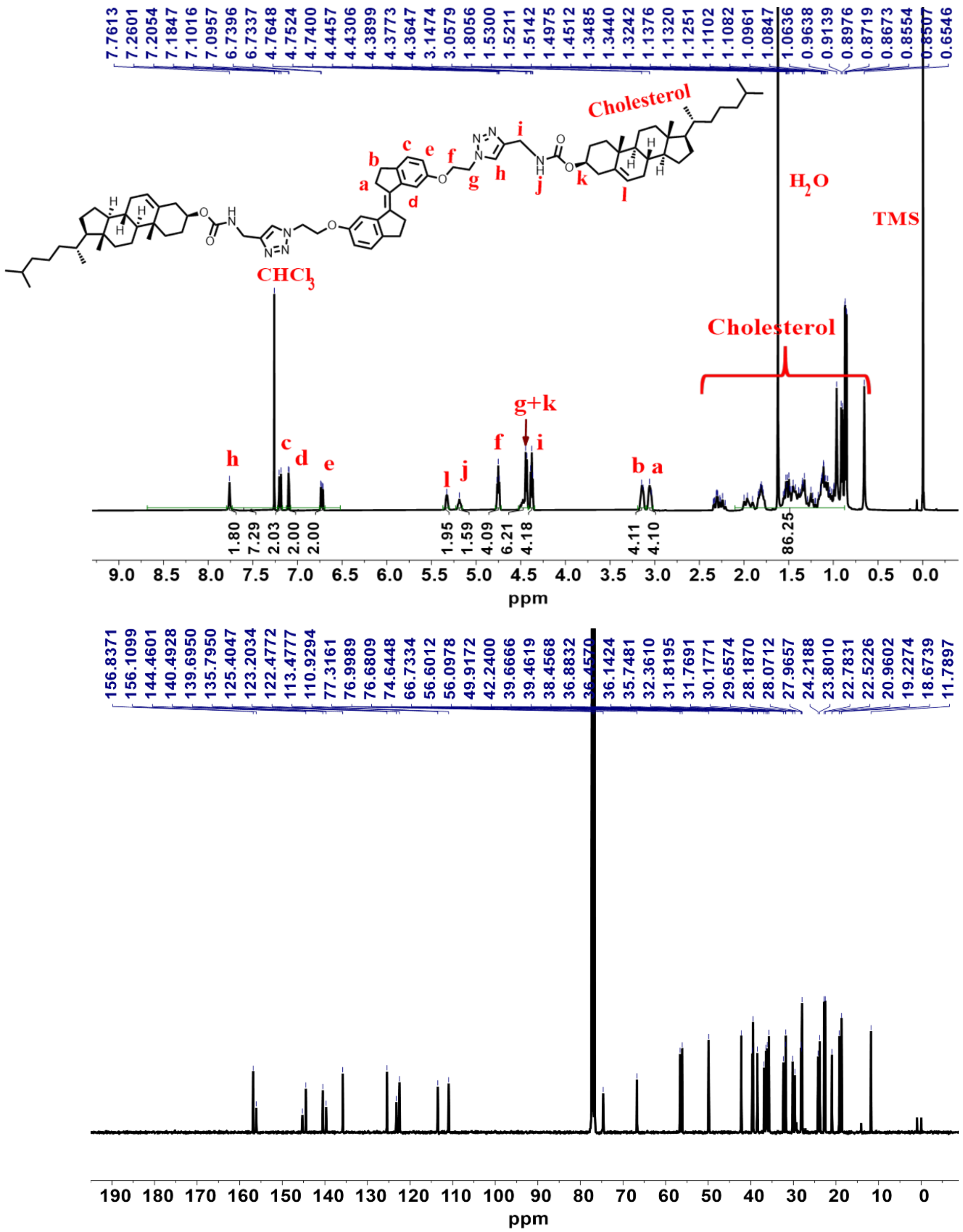

Figure S12. ^{1}H and ^{13}C NMR spectra of compound ***E*-D** in $CDCl_3$.

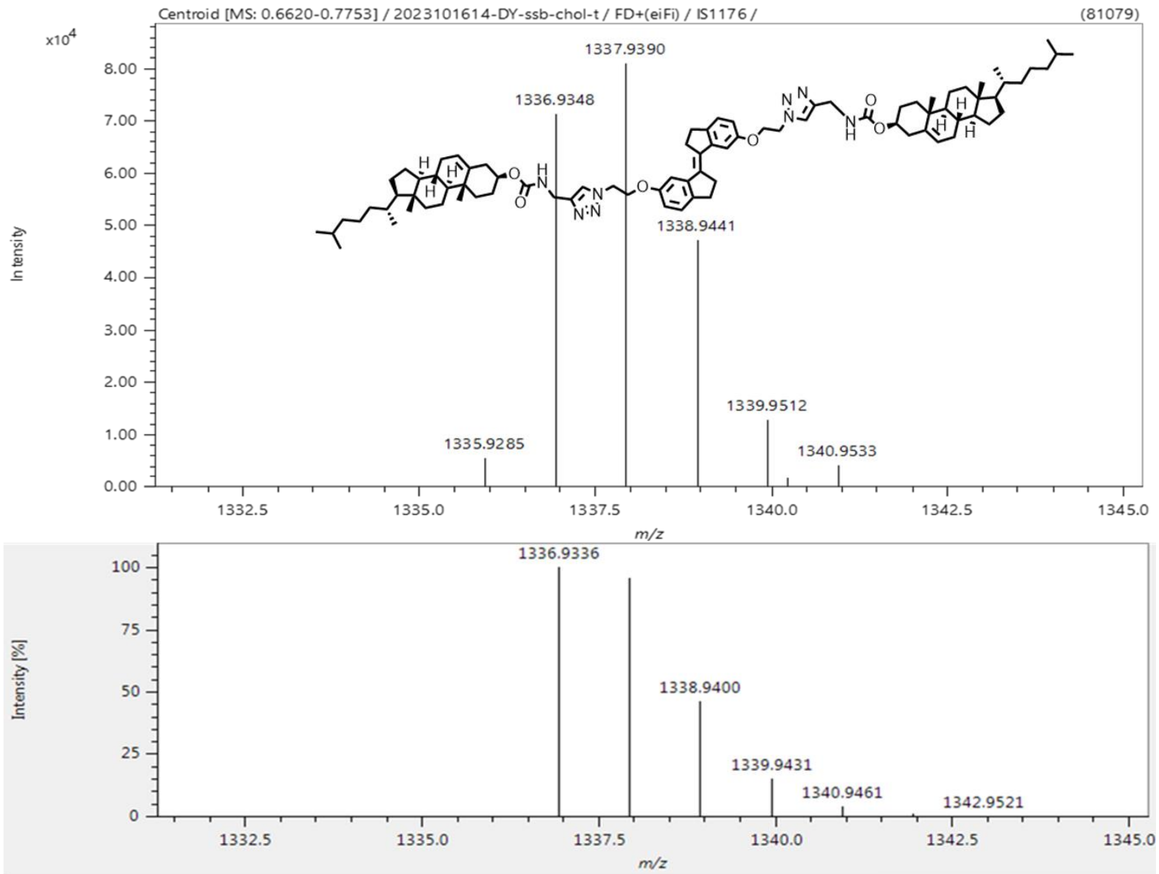

	Mass	Intensity	Formula	Calculated Mass	Mass Difference [mDa]	Mass Difference [ppm]	DBE
▸	1336.93482	71453.75	C84 H120 N8 O6	1336.93363	1.19	0.89	29.0

Figure S13. HRMS-FD (+) data of compound *E*-**D**.

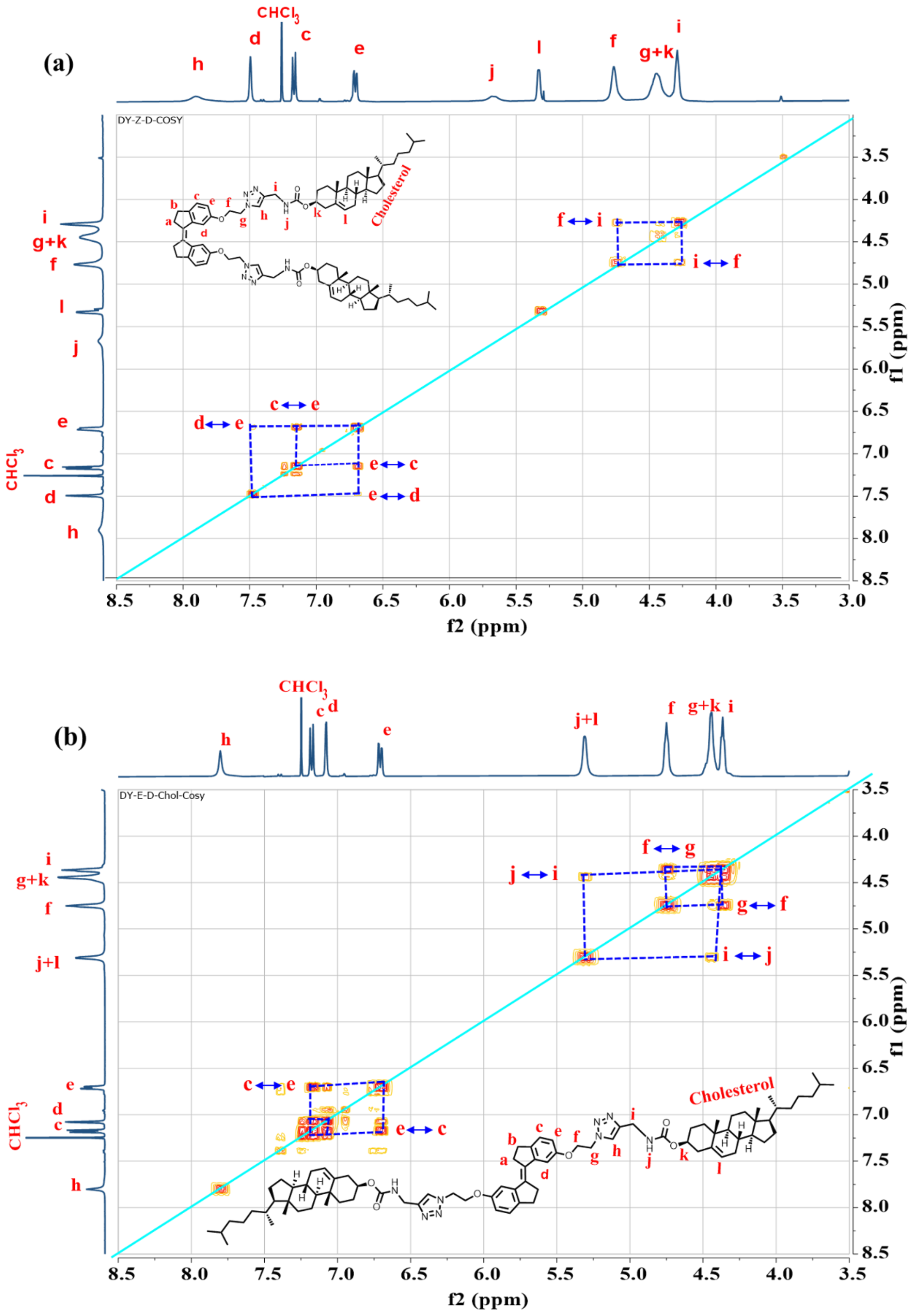

Figure S14. Partial H, H-COSY spectra of (a) *Z*-**D** and (b) *E*-**D** in $CDCl_3$.

References

S1. Alene, D. Y.; Arumugaperumal, R.; Shellaiah, M.; Sun, K. W.; Chung, W.-S., Stiff-Stilbene-Bridged Biscalix[4]arene as a Highly Light-Responsive Supramolecular Gelator. *Org. Lett.* **2021**, *23*, 2772-2776.

S2. Xu, J.-F.; Chen, Y.-Z.; Wu, D.; Wu, L.-Z.; Tung, C.-H.; Yang, Q.-Z., Photoresponsive Hydrogen-Bonded Supramolecular Polymers Based on a Stiff Stilbene Unit. *Angew. Chem., Int. Ed.* **2013**, *52*, 9738-9742.

S3. Lü, Y.; Sun, Q.; Hu, B.; Chen, X.; Miao, R.; Fang, Y., Synthesis and sensing applications of a new fluorescent derivative of cholesterol. *New J. Chem.,* **2016**, *40*, 1817-1824.
